# Destabilization of microrchidia family CW‐type zinc finger 2 via the cyclin‐dependent kinase 1‐chaperone‐mediated autophagy pathway promotes mitotic arrest and enhances cancer cellular sensitivity to microtubule‐targeting agents

**DOI:** 10.1002/ctm2.1210

**Published:** 2023-03-26

**Authors:** Shu‐Yuan Hu, Jin‐Xian Qian, Shao‐Ying Yang, Lisa Andriani, Li Liao, Ling Deng, Min‐Ying Huang, Yin‐Ling Zhang, Fang‐Lin Zhang, Zhi‐Min Shao, Da‐Qiang Li

**Affiliations:** ^1^ Shanghai Cancer Center and Institutes of Biomedical Sciences, Shanghai Medical College Fudan University Shanghai China; ^2^ Department of Breast Surgery, Shanghai Medical College Fudan University Shanghai China; ^3^ Cancer Institute, Shanghai Medical College Fudan University Shanghai China; ^4^ Department of Oncology, Shanghai Medical College Fudan University Shanghai China; ^5^ Shanghai Key Laboratory of Breast Cancer, Shanghai Medical College Fudan University Shanghai China; ^6^ Shanghai Key Laboratory of Radiation Oncology, Shanghai Medical College Fudan University Shanghai China

**Keywords:** chaperone‐mediated autophagy, cyclin‐dependent kinase 1, microtubule‐targeting agents, mitotic arrest, MORC2, spindle assembly checkpoint

## Abstract

**Background:**

Microtubule‐targeing agents (MTAs), such as paclitaxel (PTX) and vincristine (VCR), kill cancer cells through activtion of the spindle assembly checkpoint (SAC) and induction of mitotic arrest, but the development of resistance poses significant clinical challenges.

**Methods:**

Immunoblotting and RT‐qPCR were used to investigate potential function and related mechanism of MORC2. Flow cytometry analyses were carried out to determine cell cycle distribution and apoptosis. The effect of MORC2 on cellular sensitivity to PTX and VCR was determined by immunoblotting, flow cytometry, and colony formation assays. Immunoprecipitation assays and immunofluorescent staining were utilized to investigate protein‐protein interaction and protein co‐localization.

**Results:**

Here, we identified microrchidia family CW‐type zinc finger 2 (MORC2), a poorly characterized oncoprotein, as a novel regulator of SAC activation, mitotic progression, and resistance of cancer cells to PTX and VCR. Mechanically, PTX and VCR activate cyclin‐dependent kinase 1, which in turn induces MORC2 phosphorylation at threonine 717 (T717) and T733. Phosphorylated MORC2 enhances its interation with HSPA8 and LAMP2A, two essential components of the chaperone‐mediated autophagy (CMA) mechinery, resulting in its autophagic degradation. Degradation of MORC2 during mitosis leads to SAC activation through stabilizing anaphase promoting complex/cyclosome activator protein Cdc20 and facilitating mitotic checkpoint complex assembly, thus contributing to mitotic arrest induced by PTX and VCR. Notably, knockdown of MORC2 promotes mitotic arrest induced by PTX and VCR and enhances the sensitivity of cancer cells to PTX and VCR.

**Conclusions:**

Collectively, these findings unveil a previously unrecognized function and regulatory mechanism of MORC2 in mitotic progression and resistance of cancer cells to MTAs. These results also provide a new clue for developing combined treatmentstrategy by targeting MORC2 in combination with MTAs against human cancer.

## Graphical abstract

PTX and VCR promote phosphorylation of MORC2 by activating CDK1, resulting in degradation of MORC2 via the CMA pathway. Downregulation of MORC2 further activates the SAC by stabilizing Cdc20 and promoting the assembly of the mitotic checkpoint complex, thus contributing to mitotic arrest induced by PTX and VCR. Depletion of MORC2 promotes mitotic arrest and enhances cellular sensitivity to PTX and VCR.

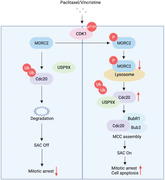

## 1 INTRODUCTION

Microtubule‐targeting agents (MTAs), such as paclitaxel (PTX) and vincristine (VCR), are effective chemotherapeutic drugs for the treatment of various malignancies.^1^ Both agents disturb the dynamic activity of microtubules, thus leading to prolonged mitotic arrest and apoptosis.^2^ In dividing cells, the microtubules that comprise the mitotic spindle are highly dynamic and extremely sensitive to MTAs. Thus, the rapid proliferative capacity of cancer cells makes them more susceptible to being targeted by these antimitotic drugs. Unfortunately, some patients become resistant to PTX and VCR prior to complete tumour eradication, and the characteristics of patients who can benefit from these drugs have not yet been determined, thus posing significant clinical challenges. Thus, it is imperative and crucial to identify the molecular characteristics of sensitivity or resistance toward PTX or VCR and select appropriate patients for this treatment based on these features.

The mechanism of prolonged mitotic arrest induced by PTX and VCR has been increasingly elucidated. When these MTAs disrupt the formation of the mitotic spindle, the spindle assembly checkpoint (SAC) is activated to arrest cells in mitosis.^3^, ^4^, ^5^ The SAC is an inherent monitoring system that ensures the accurate separation of chromosomes by delaying the transition from metaphase to anaphase until all kinetochores are appropriately attached to spindle microtubules.^6^, ^7^ During prometaphase, in response to sister chromatids improperly attaching to the mitotic spindle, the SAC is activated and promotes the assembly of the mitotic checkpoint complex (MCC), including MAD2, BubR1/Mad3 and Bub3, as well as Cdc20.^8^, ^9^ The MCC combines with and suppresses the activity of anaphase‐promoting complex/cyclosome (APC/C).^10^ Once all sister‐kinetochore pairs are properly attached to the kinetochore microtubules, Cdc20 can be separated from the MCC and activate APC/C. This results in the degradation of APC/C downstream proteins including cyclin B and securin, thereby contributing to the activation of separase that induces the segregation of sister chromatids and allows the cell to enter mitotic anaphase.^6^, ^11^, ^12^, ^13^ In typical mitosis, the SAC is activated only briefly to permit unattached chromosomes to attach to the spindle microtubules and correct misattached chromosomes. In contrast, the SAC is permanently activated in the presence of MTAs, rendering cells continuously arrested at metaphase and incapable of entering anaphase, ultimately resulting in apoptosis.^14^, ^15^ Hence, if the SAC function of cancer cells is defective or inhibited, cells can only be temporarily arrested, allowing cells to pass through mitosis without undergoing apoptosis. In practice, due to the functional deficiency of the SAC, cancer cells can resist killing by MTAs and thus increase their resistance to PTX or VCR.^14^, ^16^, ^17^

Cyclin‐dependent kinases (CDKs) are key proteins in regulating cell‐cycle progression by associating with their cyclin partners to phosphorylate their downstream substrates.^18^, ^19^ Notably, CDK1 is the only cyclin‐dependent serine/threonine protein kinase that is essential for mitotic progression.^20^ During the G2 phase, CDK1 can be phosphorylated by CDK7 at Thr161 within its T‐loop structure, thus increasing its ability to bind to its cyclin partners and activating its kinase activity.^21^, ^22^ In addition, at the G2/M boundary, CDK1 is dephosphorylated by Cdc25 at Thr14 and Tyr15 to achieve full activation.^23^ Afterward, activated CDK1, together with cyclin B, phosphorylates various substrates to drive a cell into mitosis.^24^ When a mitotic cell enters metaphase, the SAC is activated and becomes in charge of managing the exit of the cell from mitosis.^15^ Activation of the SAC inhibits the activity of APC/C, maintaining high levels of cyclin B and thereby protecting the activity of CDK1. Therefore, the activity of CDK1 gradually increases in accordance with the activation of the SAC.^16^ Moreover, mitotic arrest induced by MTAs, such as PTX and VCR, is preceded by activating CDK1.^25^, ^26^

Microrchidia family CW‐type zinc finger 2 (MORC2) is a newly identified chromatin‐remodelling enzyme and has fundamental functions in gene transcription and DNA damage repair. As a component of the human silencing hub (HUSH) complex, MORC2 affects HUSH‐mediated epigenetic gene silencing.^27^, ^28^, ^29^ In addition, recent work from our group demonstrated that MORC2 is involved in DNA damage response and contributes to the resistance of breast cancer cells to DNA‐damaging agents.^30^, ^31^, ^32^, ^33^, ^34^ Notably, mutations in MORC2 have been linked to several human diseases, including Charcot‐Marie‐Tooth disease,^35^, ^36^, ^37^, ^38^, ^39^, ^40^, ^41^, ^42^ Cockayne syndrome,^43^ neurodevelopmental disorder,^44^ motor neuropathy^45^ and triple‐negative breast cancer.^46^ Moreover, emerging evidence shows that MORC2 is upregulated in multiple types of human cancer^47^ and contributes to aggressive phenotypes of human cancer such as gastric,^48^ colorectal,^49^ liver^50^ and breast cancer.^46^, ^51^, ^52^ However, the function and regulatory mechanism of MORC2 in mitotic progression and its effect on cellular sensitivity to MTAs remain unknown.

Here, we first report that PTX and VCR induce CDK1‐mediated phosphorylation of MORC2, leading to its degradation via the chaperone‐mediated autophagy (CMA) pathway. Degradation of MORC2 upregulates Cdc20 and activates the SAC, thus contributing to prolonged mitotic arrest and apoptosis upon treatment with PTX and VCR. Moreover, knockout of endogenous MORC2 results in enhanced sensitivity of cancer cells to PTX and VCR. Overall, these discoveries reveal the novel function and related molecular mechanism of MORC2 in regulating SAC activation, mitotic progression and resistance of cancer cells to MTA‐based chemotherapy.

## 2 MATERIALS AND METHODS

### 2.1 Cell culture and treatment

Human breast cancer MCF‐7 cell line (#SCSP‐531), human embryonic kidney HEK293T cell line (#SCSP‐502) and human cervical cancer HeLa cell line (#TCHu187) were obtained from the Cell bank of Chinese Academy of Sciences (Shanghai, China), and were authenticated by detection of mycoplasma, endotoxin, isozyme, cell viability and DNA‐fingerprinting. All cell lines were maintained in high‐glucose DMEM (BasalMedia, #L110) containing 10% fetal bovine serum (ExCell Bio, #FSP500) and 1% penicillin/streptomycin (BasalMedia, #S110B) in a cell incubator at 37°C with 5% CO_2_. Unless otherwise noted, all reagents were obtained from Sigma‐Aldrich. Detailed information on chemical inhibitors is provided in Table S1.

### 2.2 Plasmids, short hairpin RNAs and small interfering RNAs

Myc‐DDK‐MORC2 cDNA (Origene, #RC200518) was inserted into lentiviral vectors pCDH‐CMV‐MCS‐EF1‐Puro and pLVX‐Neo‐IRES to generate Flag‐ and HA‐tagged MORC2 constructs. Point mutations for MORC2 were generated using PCR and the methylation‐sensitive restriction enzyme DpnI (New England Biolabs, # R0176S). To generate the HA‐Cdc20 construct, Cdc20 cDNA was amplified by PCR and ligated into lentiviral vector pLVX‐Neo‐IRES. Short hairpin RNAs (shRNAs) were synthesized, annealed and then cloned into the pLKO.1‐TRC lentiviral vector for gene silencing. DNA sequencing was performed (Sangon Biotech, Shanghai) to validate the construct sequences. The expression vectors and corresponding cloning primers are shown in Tables S2 and S3. Negative control small interfering RNA (siNC), small interfering RNAs (siRNAs) targeting heat shock protein family member 8 (HSPA8), lysosomal‐associated membrane protein type 2A (LAMP2A), CDK1 and Cdc20 were synthesized by GenePharma (Shanghai, China). SiRNA targeting sequences are presented in Table S4.

### 2.3 Plasmid transfection and lentiviral infection

Transient transfection of expression plasmids was performed using a Neofect DNA transfection reagent (TengyiBio, #TF201201). To establish stable cell lines, HEK293T cells were transfected with individual lentiviral vectors, along with packaging plasmids, to produce the viruses. After 48 h of transfection, the viruses in the supernatant were collected and filtered through 0.45 μm filter membranes. Cells were then infected with viruses supplemented with polybrene (8 μg/ml) for two days. Following lentiviral infection, puromycin (2 μg/ml) or G418 (500 μg/ml) was used to select stable cell lines. Knockout (KO) of MORC2 in HeLa and MCF‐7 cell lines was performed using the CRISPR/Cas9 system as described previously.^46^ Lipofectamine 2000/3000 Transfection Reagent (Invitrogen, #11668019 and #L3000015, respectively) was used to transfect siRNAs. The efficiency of gene overexpression or knockdown was verified by immunoblotting.

### 2.4 RNA isolation and real time‐quantitative polymerase chain reaction (RT‐qPCR) analysis

After treatment with the indicated inhibitors, cells were washed with PBS and then lysed with TRIzol reagent (Invitrogen, #15596018) for RNA extraction. After the removal of genomic DNA, mRNAs were used as templates to synthesize cDNAs using PrimeScript RT Master Mix (Vazyme, #R323‐01). The cDNAs were then used as templates for RT‐qPCR analysis using SYBR Green qPCR Master Mix (Vazyme, #Q711‐03) to detect the expression levels of target genes. GAPDH was used as an internal control. Primer sequences were obtained from PrimerBank (https://pga.mgh.harvard.edu/primerbank/) and are shown in Table S5.

### 2.5 Immunoblotting and immunoprecipitation assays

For immunoblotting assays, cells were lysed in RIPA buffer containing phosphatase and protease inhibitor cocktail (Bimake, #B15003 and #B14002). Cells were then scraped off and centrifuged to acquire the supernatant containing the protein. The same amount of protein was subsequently obtained in each sample by protein quantification. Proteins were separated via SDS‒PAGE, transferred onto PVDF membranes (Millipore, #IPVH00010) and then blocked with 5% bovine serum albumin (BSA) (Proliant Biol, #69100). Primary and secondary antibodies were then used to conjugate with proteins on the membrane, and signal detection was performed using an Enhanced Chemiluminescence Substrate Kit (Yeasen, #36208ES80). Quantitation of immunoblotting images was performed using ImageJ software. For immunoprecipitation (IP) assays, proteins were extracted from cells using NP‐40 lysis buffer. Cellular lysates were incubated with Flag‐ or HA‐tagged beads (Shanghai Genomics Technology, #GNI4510‐FG and #GNI4510‐HA‐P, respectively) on a rotator at 4°C for 3 h or with the indicated primary antibodies overnight and then with protein A/G magnetic beads (Bimake, #B23202) for another 3 h. The protein‐antibody complexes were washed thrice to remove nonspecifically bound proteins after incubation and prior to immunoblotting analysis. Detailed information about primary antibodies that were used in this study is provided in Table S6.

### 2.6 Immunofluorescent staining

Cells that grew on coverslips (Thermo Fisher Scientific, #12‐545‐80) were fixed using 4% paraformaldehyde (Sangon Biotech, #E672002‐0500) for 30 min, permeabilized with 0.5% Triton X‐100 for 15 min and then blocked using 5% BSA for 1 h. Cells were then incubated with the indicated primary antibodies overnight at 4°C, washed thrice with PBST, followed by incubation with Alexa Fluor 488 (green) (Cell Signaling Technology, #4408S or #4412S) and 555 (red) (Cell Signaling Technology, #4409S or #4413S) secondary antibodies. Fluoroshield mounting medium with DAPI (Abcam, #ab104139) was used to counterstain DNA. Fluorescence was detected using a Leica SP5 confocal laser scanning microscope (Leica Microsystems, Buffalo Grove, USA). The images were processed using PhotoShop software.

### 2.7 Cell cycle and apoptosis analysis

For cell cycle analysis, cells were collected and fixed in precooled 70% ethanol at 4°C for 2 h or overnight. After washing with PBS, cell cycle analysis was conducted with a cell cycle and apoptosis analysis kit (Yeasen, #40301ES60). For apoptosis analysis, both adherent and floating cells were harvested, washed thrice with precooled PBS and subsequently analyzed using an Annexin V‐FITC/PI Apoptosis Detection Kit (Yeasen, #40302ES60). Flow cytometry analyses were carried out on a BD FACSCanto II flow cytometer (BD Bioscience, San Jose, USA). The results were analyzed by FlowJo 10 software.

### 2.8 Cell‐cycle synchronization

To harvest cells at the G1/S boundary, a double thymidine block was performed as described previously.^53^ After synchronization, cells were released for 6 h to collect cells in the S phase. To harvest cells in the G2/M phase, the thymidine‐nocodazole arrest was performed as described previously.^53^ To collect cells in mitosis, cells were shaken off after being synchronized by thymidine‐nocodazole arrest. Flow cytometry analysis was performed after cell collection to determine cell‐cycle distribution.

### 2.9 Colony formation assays

Cells were digested, counted and seeded into 6‐ or 12‐well plates (1000 cells/well) in triplicate. After being incubated overnight, cells were then exposed to the indicated drugs for 10–14 days. The survival colonies were then washed with PBS, fixed in methanol, stained with 1% crystal violet and counted.

### 2.10 In vivo ubiquitination assays

In vivo ubiquitination assays were carried out as described previously.^54^ In brief, transfection of the indicated expression plasmids individually or in combination was conducted in HEK293T cells. After 48 h of transfection, cells were incubated with 10 μM MG‐132 for 6 h, lysed with the denaturing solution and analyzed via IP analysis to determine protein ubiquitination levels.

### 2.11 Statistical analysis

Quantification and statistical analysis were performed using FlowJo 10 software (version 10.8.1), ImageJ (version 1.8.0) and GraphPad (version 8.0.2). The means and standard deviations of at least three independent experiments are presented. Comparisons between the two groups were evaluated using the two‐tailed Student's *t*‐test. *P‐*Value less than 0.05 was considered statistically significant (*, *p* < 0.05; **, *p* < 0.01; ***, *p* < 0.001; ns, no significance).

## 3 RESULTS

### 3.1 MORC2 protein levels are decreased following treatment of cells with PTX and VCR

**FIGURE 1:**
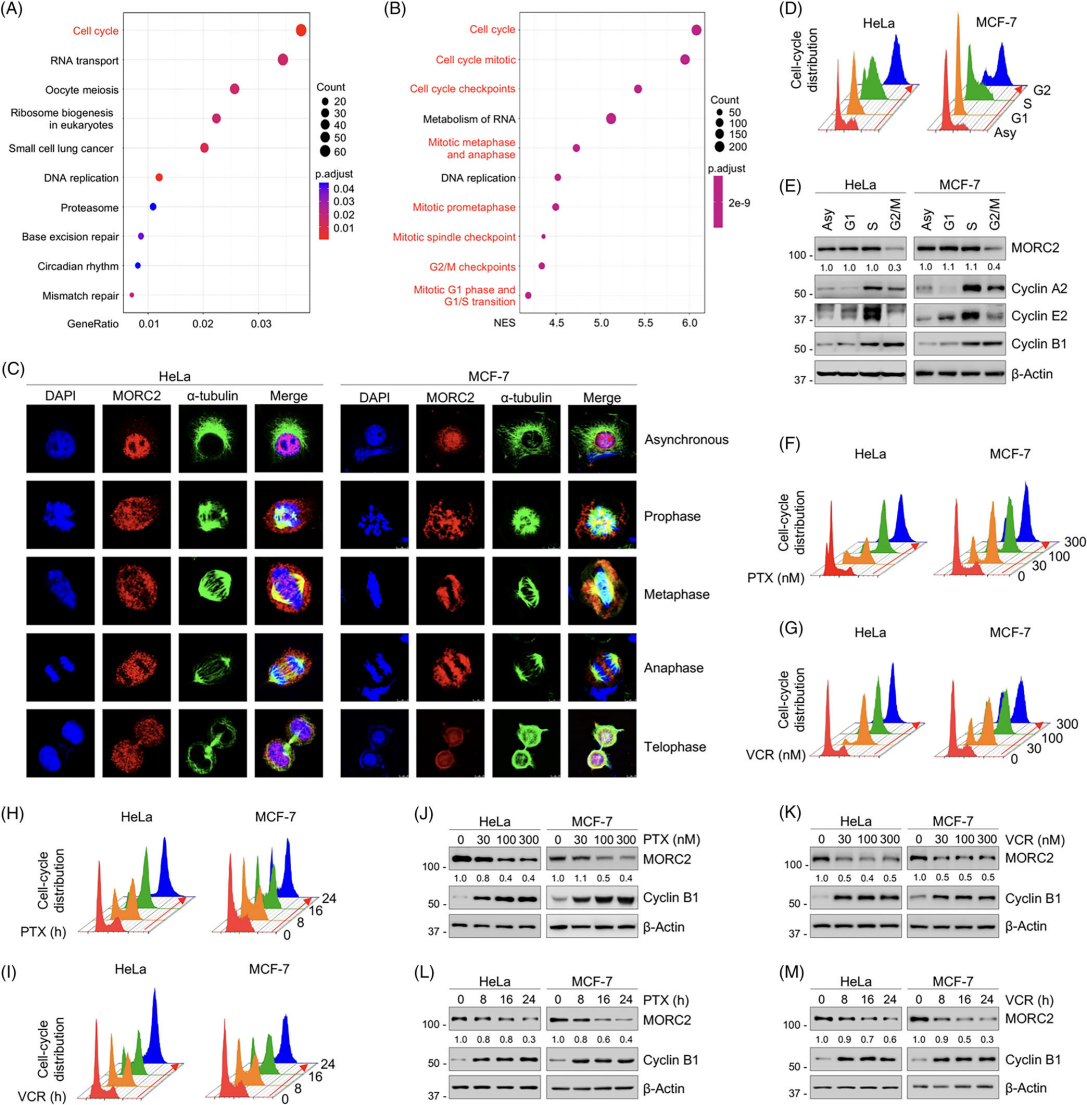
MORC2 protein levels are decreased following treatment of cells with PTX and VCR. (A, B) KEGG (A) and GSEA (B) analyses using TCGA breast cancer database. (C) Immunofluorescent staining was performed to detect alternations in subcellular localization of MORC2. HeLa and MCF‐7 cells were stained with an anti‐MORC2 (red) or an α‐tubulin (green) antibody. DAPI (blue) was used to stain DNA. (D) HeLa and MCF‐7 cells were synchronized at the indicated stages of the cell cycle through the double‐thymidine block and thymidine‐nocodazole arrest. Flow cytometric analysis was performed to determine cell‐cycle distribution. (E) HeLa and MCF‐7 cells were synchronized at the indicated stages of the cell cycle through the double‐thymidine block and thymidine‐nocodazole arrest. Immunoblotting analysis was performed to detect MORC2 protein levels. (F, G) HeLa and MCF‐7 cells were treated with the indicated doses of PTX (F) or VCR (G) for 24 h.Flow cytometric analysis was performed to determine cell‐cycle distribution. (H, I) HeLa and MCF‐7 cells were treated with 100 nM PTX (H) or VCR (I) for the indicated times. Flow cytometric analysis was performed to determine cell‐cycle distribution. (J, K) HeLa and MCF‐7 cells were treated with the indicated doses of PTX (J) or VCR (K) for 24 h. Immunoblotting analysis was performed to detect MORC2 protein levels. (L, M) HeLa and MCF‐7 cells were treated with 100 nM PTX (L) or VCR (M) for the indicated times. Immunoblotting analysis was performed to detect MORC2 protein levels.

To investigate the novel function and related mechanism of MORC2, we first conducted Kyoto Encyclopedia of Genes and Genomes (KEGG) analysis (Figure 1A) and gene set enrichment analysis (GSEA) (Figure 1B) using The Cancer Genome Atlas (TCGA) breast cancer database (http://guotosky.vip:13838/GTBA/). A significant correlation was found between the expression levels of MORC2 and the cell‐cycle pathway (Figure 1A,B). As demonstrated by immunofluorescent staining, MORC2 was predominantly localized in the nucleus during interphase. In contrast, MORC2 was translocated to the cytoplasm from the nucleus and co‐localized with microtubule protein α‐tubulin during mitosis progression, especially in prophase, metaphase and anaphase (Figure 1C). These results suggest that MORC2 may be regulated during cell‐cycle progression. We validated this hypothesis by synchronizing HeLa and MCF‐7 cells at each stage of the cell cycle (Figure 1D) and then carrying out immunoblotting analysis with cell‐cycle marker antibodies. We found that MORC2 expression levels were reduced in the G2/M phase, indicating that MORC2 protein levels may be regulated during the cell cycle, especially in the mitotic phase (Figure 1E).

Given that PTX and VCR work by blocking cells in mitosis and the localization and expression levels of MORC2 were altered during mitosis, we proceeded to investigate whether MORC2 protein levels would be affected by PTX and VCR. Both drugs could indeed block cells in mitosis in a dose‐ and time‐dependent manner, as determined by flow cytometry analysis (Figure 1F–I). We discovered that both drugs downregulated the expression levels of MORC2 (cyclin B1 was used as a positive control) (Figure 1J–M). Overall, these results suggest that the mitotic cells induced by PTX and VCR tend to express lower levels of MORC2.

### 3.2 PTX and VCR induce MORC2 degradation via the CMA pathway

To address the mechanism by which PTX and VCR induce MORC2 downregulation, we first examined whether both drugs regulate MORC2 transcriptionally. As confirmed by RT‐qPCR assays, PTX or VCR treatment had no noticeable effect on MORC2 mRNA levels (Figure S1A–D). Thus, protein degradation may be responsible for the decrease in MORC2 expression levels in response to drug treatment.

**FIGURE 2:**
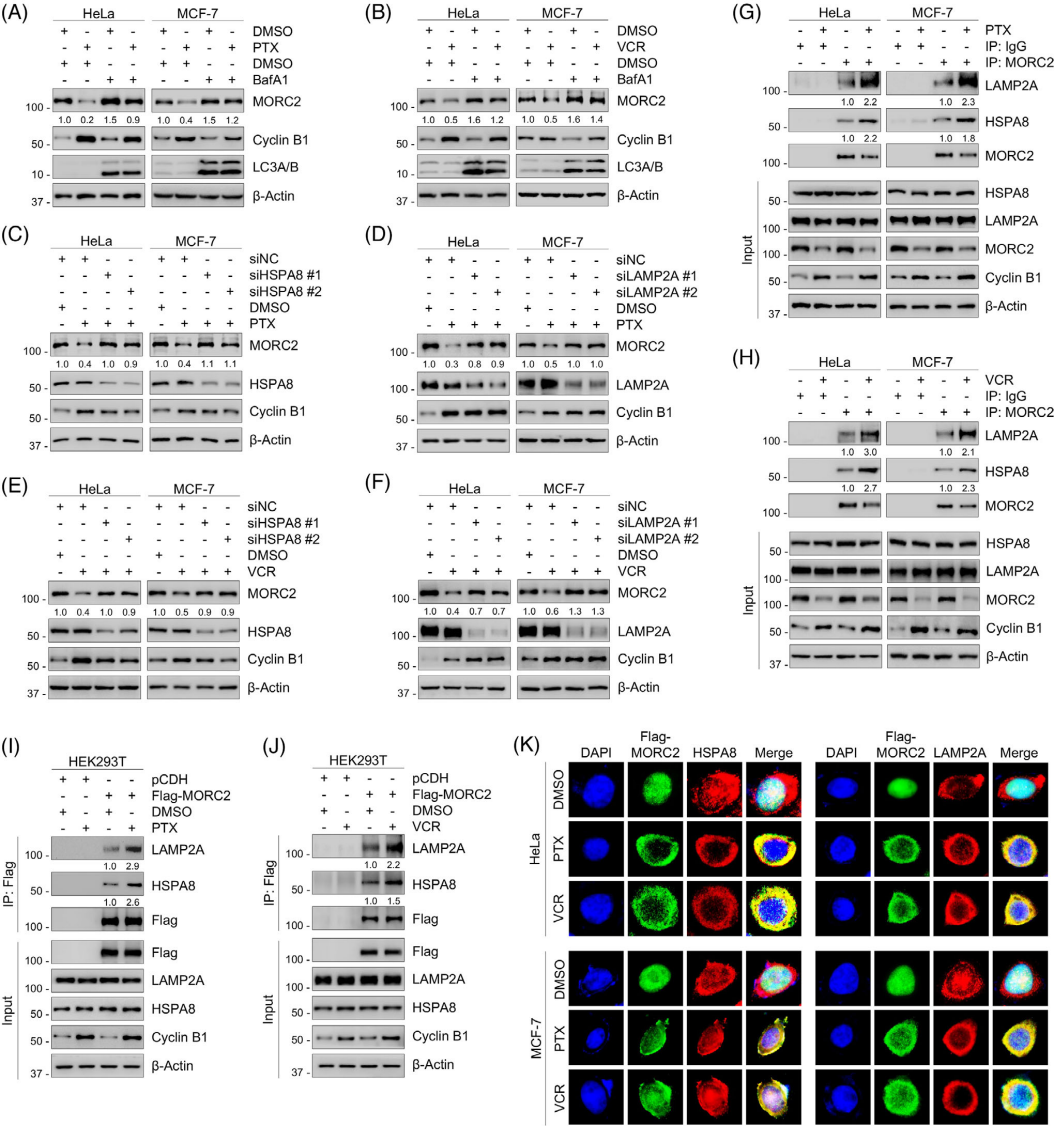
PTX and VCR induce MORC2 degradation via the CMA pathway. (A, B) HeLa and MCF‐7 cells were preincubated with or without 50 ng/ml bafilomycin A1 (BafA1) for 1 h and then treated with or without 100 nM PTX (A) or VCR (B) for 24 h. Immunoblotting analysis was performed to detect MORC2 protein levels. (C, D) HeLa and MCF‐7 cells were transfected with negative control siRNA (siNC) or siRNAs targeting HSPA8 (siHSPA8) (C) or LAMP2A (siLAMP2A) (D), followed by treatment with DMSO or 100 nM PTX for 24 h. Immunoblotting analysis was performed to detect MORC2 protein levels. (E, F) HeLa and MCF‐7 cells were transfected with siNC or siHSPA8 (E) or siLAMP2A (F), followed by treatment with DMSO or 100 nM VCR for 24 h. Immunoblotting analysis was performed to detect MORC2 protein levels. (G, H) HeLa and MCF‐7 cells were treated with or without 100 nM PTX (G) or VCR (H) for 24 h. IP analysis was performed using control IgG or an anti‐MORC2 antibody to detect the interactions between MORC2 and HSPA8/LAMP2A. (I, J) HEK293T cells were transfected with pCDH or Flag‐MORC2 expression vector, followed by treatment with or without 100 nM PTX (I) or VCR (J) for 24 h. IP analysis using Flag‐tagged beads was performed to detect the interactions between exogenous MORC2 and HSPA8/LAMP2A. (K) HeLa and MCF‐7 cells were transfected with Flag‐MORC2 expression vector, followed by treatment with or without 100 nM PTX or VCR for 24 h. Immunofluorescent staining was performed to detect the co‐localization between MORC2 and HSPA8/LAMP2A. Cells were stained with an anti‐Flag (green) or HSPA8 (red) or LAMP2A (red) antibody. DAPI (blue) was used to stain DNA.

Since proteasome and lysosome are the primary proteolytic machinery responsible for protein degradation in cells, we then tested whether MORC2 reduction following PTX or VCR treatment is due to degradation by the ubiquitin‐proteasome pathway or lysosomal proteolysis. Examination of the amino acid sequence of human MORC2 found that MORC2 contains a conserved and canonical destruction box (D‐box) motif (Figure S2A). Numerous proteins containing this motif, such ascyclin B1 and Nek2A, are targeted by the APC/C complex and degraded through the ubiquitin‐proteasome pathway.^55^ Nevertheless, the levels of MORC2 protein in HeLa and MCF‐7 cells were not dramatically affected by treatment with proteasomal inhibitor MG‐132 (Figure S2B). As a positive control, a remarkable increase in p21 protein levels was observed in these assays (Figure S2B). Moreover, incubation of HeLa and MCF‐7 cells with MG‐132 did not rescue the downregulation of MORC2 induced by PTX and VCR (Figure S2C,D), suggesting that MORC2 degradation induced by PTX and VCR is not mediated by the ubiquitin‐proteasome pathway. In contrast, treatment with the lysosomal inhibitor bafilomycin A1 (BafA1) facilitated the accumulation of MORC2 protein (Figure S2E) and was able to restore downregulated MORC2 induced by PTX and VCR (LC3A/B was used as a positive control for the autophagy degradation system) (Figure 2A,B), suggesting that MORC2 may be degraded via the autophagy‐lysosome pathway. Based on our previous studies,^56^ we speculated that MORC2 may be degraded through the CMA pathway. During the CMA process, HSPA8 functions in recognizing the substrates and translocating them to the lysosomal surface. The substrates are subsequently transported by LAMP2A to the lumen of the lysosome for degradation.^57^ To test whether MORC2 protein levels are modulated by the CMA pathway, we knocked down endogenous HSPA8 and LAMP2A using specific siRNAs. It was found that knockdown of HSPA8 and LAMP2A increased MORC2 protein levels (Figure S2F,G) and partially restored the reduced MORC2 levels induced by PTX and VCR (Figure 2C–F). Furthermore, as confirmed by IP assays, the interaction of endogenous or exogenous MORC2 with HSPA8/LAMP2A was increased in the presence of PTX and VCR (Figure 2G–J). Immunofluorescent staining revealed that PTX and VCR translocated MORC2 from the nucleus into the cytoplasm and increased its co‐localization with HSPA8 and LAMP2A (indicated by the yellow colour in the merged images) (Figure 2K). Based on these findings, we speculated that the CMA pathway may be involved in MORC2 degradation in response to PTX and VCR.

### 3.3 Downregulation of MORC2 by PTX and VCR is dependent on CDK1

**FIGURE 3:**
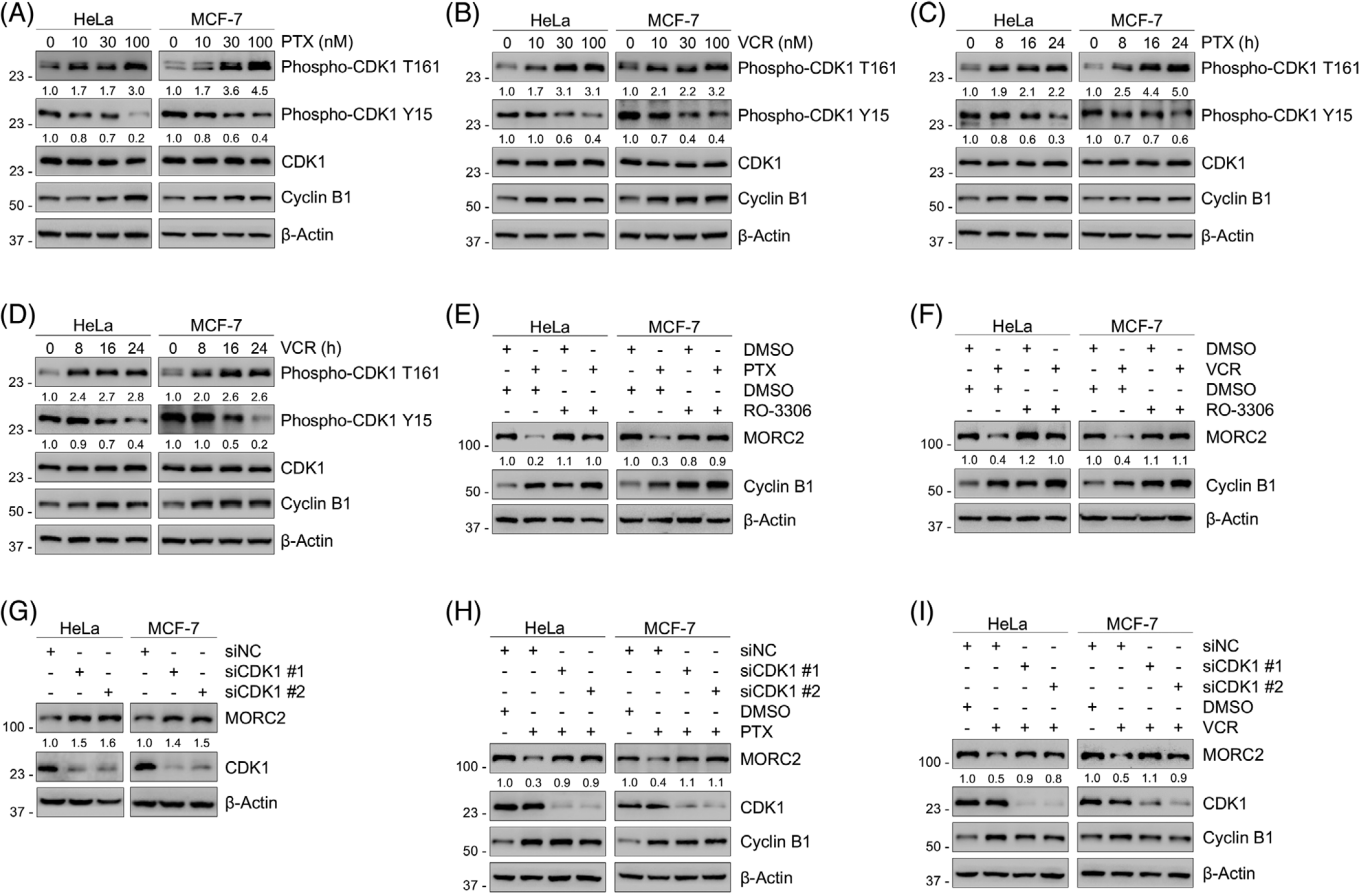
Downregulation of MORC2 by PTX and VCR is dependent on CDK1. (A‐B) HeLa and MCF‐7 cells were treated with indicated doses of PTX (A) or VCR (B) for 24 h. Immunoblotting analysis was performed to detect the phosphorylation status of CDK1. (C‐D) HeLa and MCF‐7 cells were treated with 100 nM PTX (C) or VCR (D) for the indicated times. Immunoblotting analysis was performed to detect the phosphorylation status of CDK1. (E‐F) HeLa and MCF‐7 cells were pretreated with 5 μM RO‐3306 for 1 h and then incubated with or without 100 nM PTX (E) or VCR (F) for 24 h. Immunoblotting analysis was performed to detect MORC2 protein levels. (G) HeLa and MCF‐7 cells were transfected with siNC or siRNAs targeting CDK1 for 48 h. Immunoblotting analysis was performed to detect MORC2 protein levels. (H‐I) HeLa and MCF‐7 cells were transfected with siNC or siRNAs targeting CDK1 and then treated with or without 100 nM PTX (H) or VCR (I) for 24 h. Immunoblotting analysis was performed to detect MORC2 protein levels.

Protein phosphorylation and proteolysis are the two main posttranslational mechanisms that play crucial roles in the regulation of mitosis progression.^58^ Especially, CDK1 functions as the master regulator by forming a complex with cyclin B during mitosis.^59^ Immunoblotting analysis showed these two drugs increased CDK1 phosphorylation at Thr‐161 and decreased CDK1 phosphorylation at Try‐15, indicating that CDK1 was activated in response to PTX and VCR treatment (Figure 3A–D). We further examined whether CDK1 activation is conducive to MORC2 degradation. As shown in Figure 3E,F, MORC2 degradation induced by PTX or VCR was compromised by pretreatment withCDK1 inhibitor RO‐3306. In contrast, pretreatment with PLK1 inhibitor BI‐2536, Aurora A/B inhibitor VX‐680, or CK2 inhibitor CX‐4945 had no significant effect on MORC2 degradation induced by PTX (Figure S3A–C). These results collectively suggest that MORC2 degradation induced by PTX or VCR is mediated by CDK1. Furthermore, knockdown of CDK1 using siRNAs led to an increase in MORC2 protein levels (Figure 3G) and partially rescued downregulated MORC2 following PTX and VCR treatment (Figure 3H, I). These data demonstrated that CDK1 is essential for PTX‐ and VCR‐induced MORC2 degradation.

### 3.4 CDK1 mediates MORC2 phosphorylation in response to PTX and VCR treatment

**FIGURE 4:**
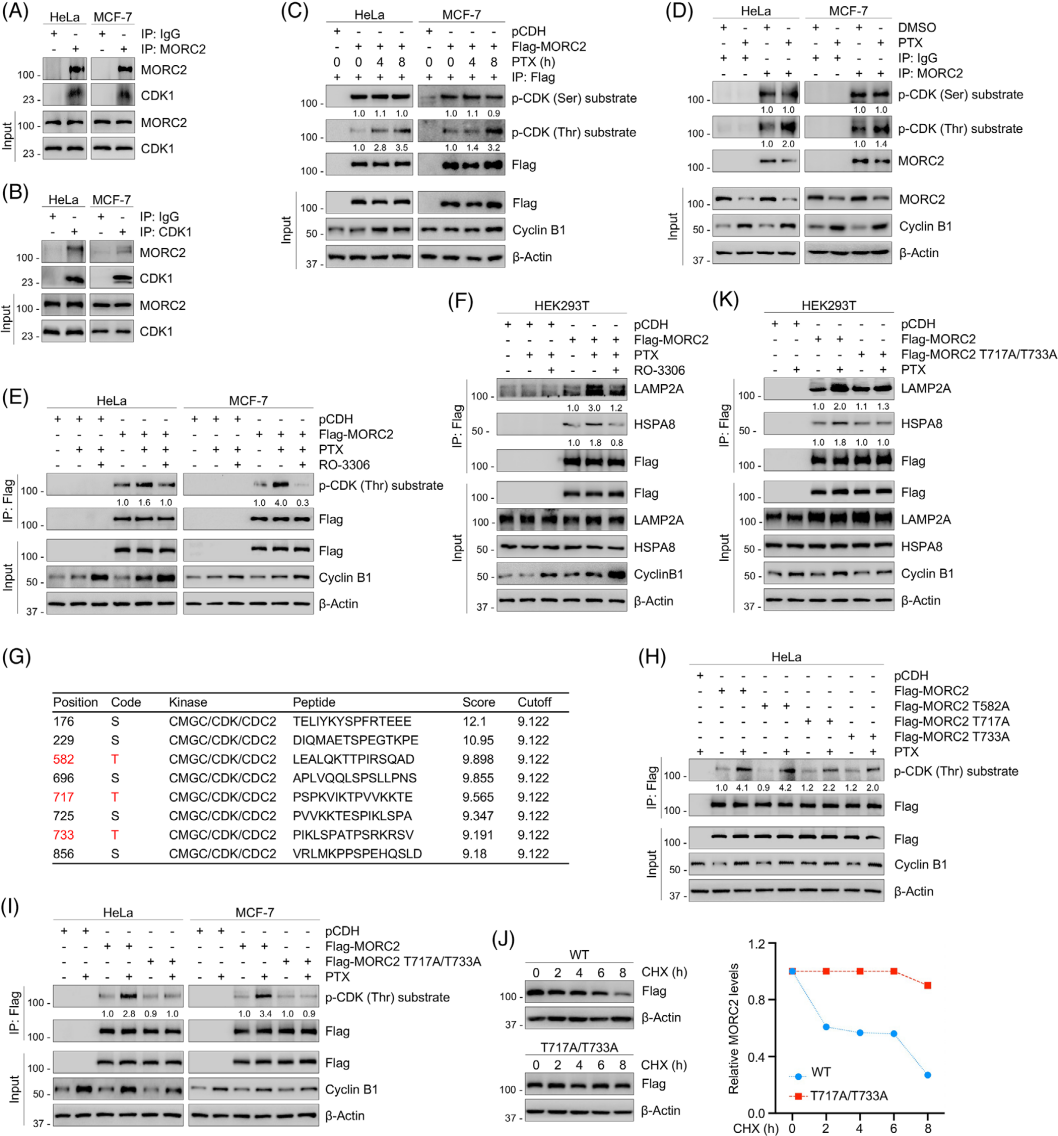
PTX and VCR promote CDK1‐mediated phosphorylation of MORC2 to induce its degradation. (A) IP assays using control IgG or an anti‐MORC2 antibody were performed to detect the interactions between MORC2 and CDK1 in HeLa and MCF‐7 cells. (B) IP assays using control IgG or an anti‐CDK1 antibody were performed to detect the interactions between CDK1 and MORC2 in HeLa and MCF‐7 cells. (C) HeLa and MCF‐7 cells were transfected with pCDH or Flag‐MORC2 expression vector and then treated with 100 nM PTX for the indicated times. IP assays using Flag‐tagged beads were performed to detect the phosphorylation levels of MORC2. (D) HeLa and MCF‐7 cells were treated with DMSO or 100 nM PTX for 24 h. IP assays using control IgG or an anti‐MORC2 antibody were performed to detect the phosphorylation levels of MORC2. (E) HeLa and MCF‐7 cells were transfected with pCDH or Flag‐MORC2 expression vector, pretreated with 5 μM RO‐3306 for 1 h, followed by incubation with or without 100 nM PTX for 24 h. IP assays using Flag‐tagged beads were performed to detect the phosphorylation levels of MORC2. (F) HEK293T cells were transfected with pCDH or Flag‐MORC2 expression vector, pretreated with 5 μM RO‐3306 for 1 h, followed by incubation with or without 100 nM PTX for 24 h. IP assays using Flag‐tagged beads were performed to detect the interactions between exogenous MORC2 and endogenous HSPA8/LAMP2A. (G) The group‐based prediction system (GPS) 5.0 software (http://gps.biocuckoo.org) was used to predict CDK1‐specific phosphorylation sites in MORC2. (H) HeLa cells were transfected with pCDH, WT and mutant Flag‐MORC2 expression vectors, followed by treatment with or without 100 nM PTX for 24 h. IP assays using Flag‐tagged beads were performed to detect the phosphorylation levels of MORC2. (I) HeLa and MCF‐7 cells were transfected with pCDH, WT and T717A/T733A mutant Flag‐MORC2 expression vectors, followed by treatment with or without 100 nM PTX for 24 h. IP assays using Flag‐tagged beads were performed to detect the phosphorylation levels of MORC2. (J) HEK293T cells were transfected with WT and T717A/T733A mutant Flag‐MORC2 expression vectors, preincubated with 100 nM PTX for 1 h and then incubated with 100 μg/ml CHX for the indicated times. Immunoblotting analysis was performed to detect the stability of MORC2 protein. (K) HEK293T cells were transfected with pCDH, WT and T717A/T733A mutant Flag‐MORC2 expression vectors, followed by treatment with or without 100 nM PTX for 24 h. IP assays using Flag‐tagged beads were performed to detect the interactions between exogenous MORC2 and endogenous HSPA8/LAMP2A.

Since CDK1 is a serine/threonine kinase that drives cell‐cycle progression by phosphorylating its downstream substrates, we further explored whether CDK1 could induce the phosphorylation of MORC2. Reciprocal IP assays showed that MORC2 interacted with CDK1 (Figure 4A,B). We next investigated whether CDK1 could induce MORC2 phosphorylation at serine or threonine residues during PTX‐ or VCR‐induced mitotic arrest via exogenous and endogenous IP analysis. Phosphorylation levels of MORC2 were determined using a phospho‐CDK substrate antibody that specifically recognizes phosphorylated serine or threonine residues by CDK1. Results showed that treatment of cells with either PTX or VCR significantly increased the phosphorylation of MORC2 at threonine residues, while the phosphorylation levels of MORC2 at the serine residues remained unchanged (Figure 4C,D and Figure S4A,B). Moreover, pretreatment with CDK1 inhibitor RO‐3306 abolished the increase in phosphorylation levels of MORC2 at threonine residues induced by PTX or VCR (Figure 4E and Figure S4C). We next tested whether phosphorylation of MORC2 is critical for its degradation. IP assays showed that treatment of cells with CDK1 inhibitor RO‐3306 abolished the increase in the interaction of MORC2 with HSPA8 and LAMP2A in response to PTX and VCR treatment (Figure 4F and Figure S4D). Conclusively, these results demonstrated that PTX and VCR promote MORC2 degradation via the CMA pathway by inducing CDK1‐dependent MORC2 phosphorylation.

### 3.5 Phosphorylation of MORC2 at T717 and T733 is required for its degradation

We next predicted potential CDK1‐specific phosphorylation sites in MORC2 using a group‐based prediction system (GPS) version 5.0 (http://gps.biocuckoo.org) and identified three potent threonine phosphorylation sites, including threonine 582 (T582), T717 and T733 (Figure 4G). All three sites accord with the proline‐directed consensus sequence of CDK1 phosphorylation sites. To further verify these results, three potential phosphorylation residues in MORC2 were individually mutated to alanine (termed T582A, T717A and T733A, respectively). Then, wild‐type (WT) MORC2 or these mutants were transfected into HeLa cells and the phosphorylation state of MORC2 was detected using IP assays. Interestingly, only the T717A and T733A mutations slightly decreased MORC2 phosphorylation levels upon treatment with PTX compared to WT and T582A mutant MORC2 (Figure 4H). In addition, a sequence alignment revealed that the T717 and T733 residues are highly conserved across various species (Figure S4E,F), indicating that both sites may be evolutionarily and functionally important. Since both T717 and T733 sites have been identified as potential mitotic phosphorylation sites of MORC2 in large‐scale proteomic investigations,^60^, ^61^, ^62^ we then mutated both T717 and T733 residues to alanine (termed T717A/T733A). Notably, mutating both residues significantly attenuated CDK1‐mediated MORC2 phosphorylation induced by PTX or VCR (Figure 4I and Figure S4G) and enhanced the stability of MORC2 protein (Figure 4J). Consistently, mutating both T717 and T733 sites significantly abolished the increase in the interaction of MORC2 with LAMP2A/HSPA8 following PTX or VCR treatment (Figure 4K and Figure S4H). Collectively, these results demonstrated that MORC2 is phosphorylated by CDK1 at T717 and T733 in response to PTX and VCR treatment, thereby promoting its subsequent lysosomal degradation via the CMA pathway.

### 3.6 Depletion of MORC2 enhances cellular sensitivity to PTX and VCR

**FIGURE 5:**
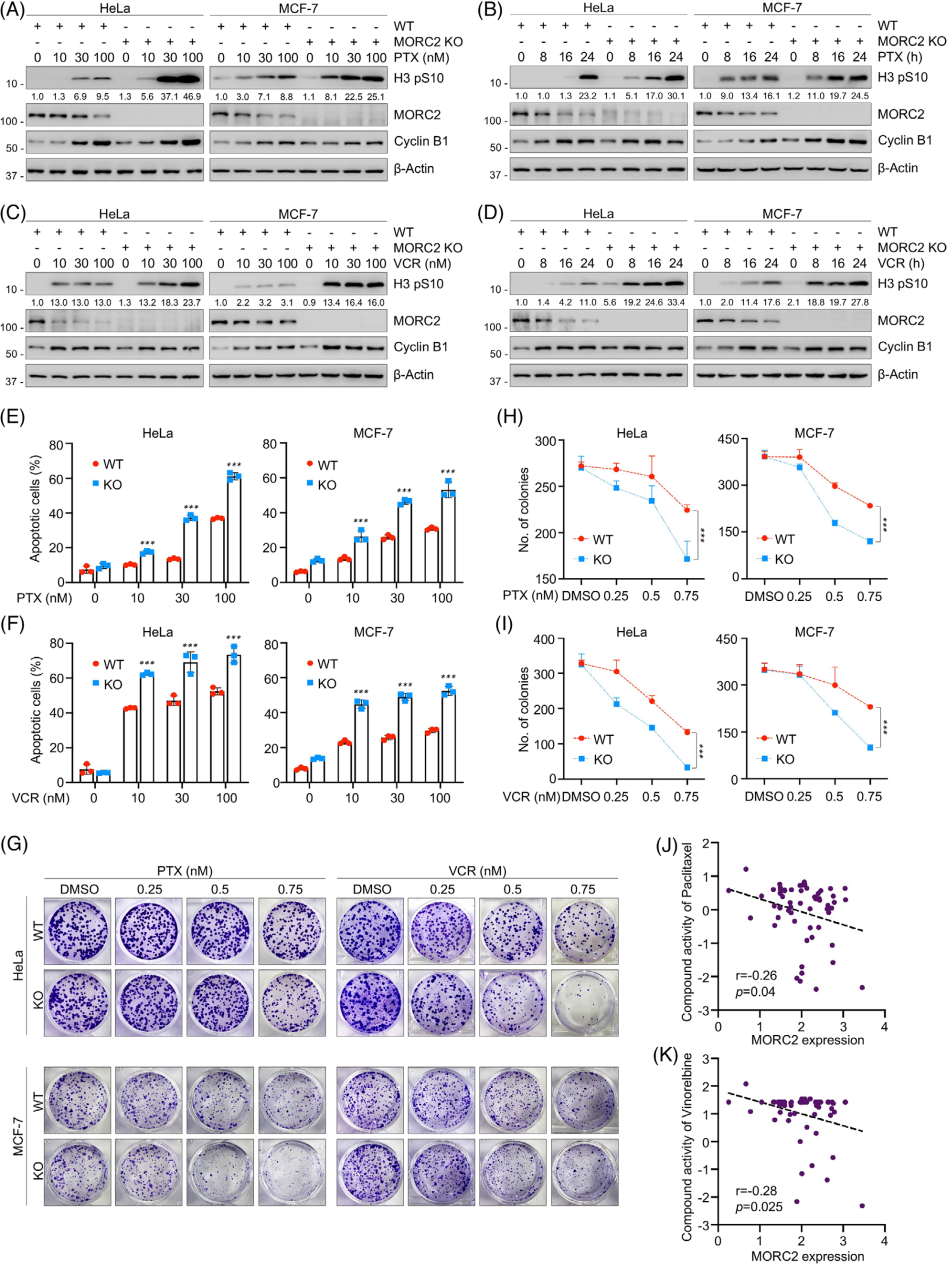
Depletion of MORC2 enhances the sensitivity of cancer cells to PTX and VCR. (A, B) WT and MORC2‐KO HeLa and MCF‐7 cells were treated with indicated doses of PTX for 24 h (A) or with 100 nM PTX for the indicated times (B). Immunoblotting analysis was performed to detect the expression levels of H3 pS10. (C, D) WT and MORC2‐KO HeLa and MCF‐7 cells were treated with 100 nM VCR for 24 h (C) or with 100 nM VCR for the indicated times (D). Immunoblotting analysis was performed to detect the expression levels of H3 pS10. (E, F) WT and MORC2‐KO HeLa and MCF‐7 cells were treated with indicated doses of PTX (E) or VCR (F) for 48 h. Apoptosis analysis via flow cytometry was performed to detect the percentage of cells that undergo apoptosis in response to drug treatment. (G–I) WT and MORC2‐KO HeLa and MCF‐7 cells were treated with PTX or VCR at indicated concentrations and subjected to colony formation assays. After 10−14 days of treatment, survival colonies were stained with 1% crystal violet and counted. Representative images of survival colonies are presented in G, and the corresponding quantitative results are shown in H and I. (J, K) The correlation between MORC2 expression levels and drug activity of PTX (J) and vinorelbine (K) was analyzed using the CellMiner database.

Given that the main antitumor effects of PTX and VCR are mediated by inducing mitotic arrest and that PTX and VCR promote MORC2 degradation, we next questioned whether MORC2 knockdown in combination with PTX and VCR has a further impact on mitotic arrest and apoptosis. To address this question, WT and MORC2‐knockout (KO) HeLa and MCF‐7 cells were subjected to PTX and VCR treatment. Immunoblotting analyses showed that expression levels of histone H3 phosphorylated at Ser10 (H3 pS10), a mitotic marker,^63^ in MORC2‐KO cells were markedly higher than those in control cells (Figure 5A–D), indicating that a greater proportion of cells were arrested in mitosis after MORC2 knockout. Flow cytometric analysis also demonstrated that knockout of MORC2 resulted in a larger percentage of cells in the G2/M phase after treatment with PTX and VCR (Figure S5). As these antimitotic chemotherapeutic agents arrest cell‐cycle progression to impel cancer cells to undergo apoptosis, we further conducted apoptosis assays via flow cytometry. Results showed that knockout of MORC2 enhanced PTX‐ or VCR‐induced apoptosis (Figure 5E,F and Figure S6A). Colony formation assays further confirmed that MORC2‐KO cells were more sensitive to PTX or VCR treatment (Figure 5G–I). Moreover, we assessed whether a correlation existes between drug activity of PTX/VCR and expression levels of MORC2 using the CellMiner database,^64^ which contains drug sensitivity information for 60 cancer cell lines. It was found that MORC2 expression levels were negatively correlated with the compound activity of PTX, further confirming that high expression of MORC2 could reduce the sensitivity of cancer cells to PTX (Figure 5J). Although the expression levels of MORC2 also had a negative trend with the drug activity of VCR, it was not statistically significant (Figure S6B). Nevertheless, we found that high expression of MORC2 could lead to low activity of vinorelbine, a derivative of VCR that also works by blocking cells in mitosis (Figure 5K). In summary, these results suggest that the downregulation of MORC2 synergistically enhances the growth inhibitory effects of PTX and VCR.

To further determine the role of MORC2 downregulation on mitotic arrest induced by PTX and VCR, we reintroduced WT or T717A/T733A mutant MORC2 into endogenous MORC2‐KO cells by lentiviral infection (Figure S7A). Consistent with the above results, we found that T717A/T733A mutant MORC2 was more stable than its WT counterpart in the presence of PTX and VCR (Figure S7B,C). Moreover, T717A/T733A mutant‐expressing cells had lower levels of H3 pS10 after PTX and VCR treatment, suggesting that the more stabilized MORC2 further impairs mitotic arrest (Figure S7B,C). Hence, the foregoing data suggest that mitotic arrest induced by PTX and VCR is, at least in part, dependent on MORC2 degradation. Thus, the effect of these drugs on the mitotic arrest can be enhanced by the inhibition of MORC2 levels.

Moreover, we next examined whether MORC2 depletion could affect the sensitivity of non‐transformed cells to PTX and VCR. We knocked down MORC2 in mammary epithelial MCF‐10A cells and performed immunoblotting and cell cycle analysis. To our surprise, MORC2 depletion had no significant impact on H3 pS10 expression levels or the percentage of cells blocked in G2/M after PTX or VCR treatment (Figure S8A–D). In addition, as demonstrated by CCK‐8 and colony formation assays, MORC2 depletion also had no remarkable effect on cellular sensitivity of MCF10A cells to PTX and VCR (Figure S8E–H). Collectively, these findings suggest that knockdown of MORC2 in non‐transformed cells does not enhance the effects of PTX and VCR.

### 3.7 MORC2 compromises the SAC function

As mentioned above, MORC2 has been reported to interact with the HUSH complex, participate in chromatin compaction and suppress the transcription of LINE‐1 elements.^27^ Since LINE‐1 components are directly associated with apoptosis,^65^, ^66^ we verified whether LINE‐1s are overexpressed in PTX‐ and VCR‐treated cells. However, RT‐qPCR assays demonstrated that the expression levels of LINE‐1s did not change significantly upon drug treatment (Figure S9). These results indicate that MORC2 degradation and subsequent apoptosis induced by PTX and VCR are not due to the derepression of LINE‐1 elements.

**FIGURE 6:**
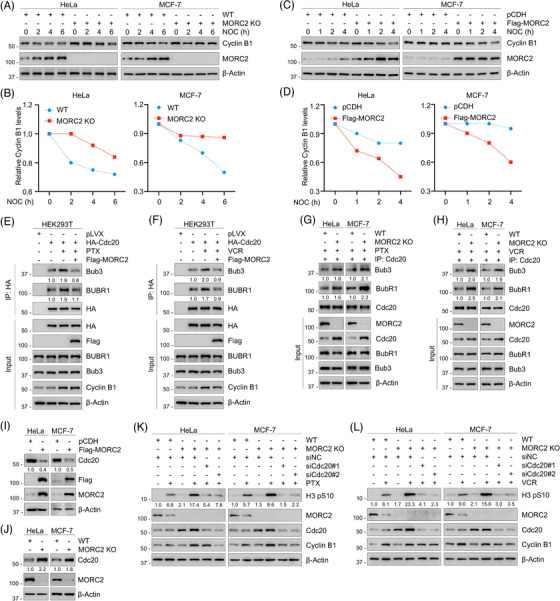
MORC2 compromises the SAC function. (A, B) WT and MORC2‐KO HeLa and MCF‐7 cells were synchronized at prometaphase using nocodazole and released into mitosis for the indicated times. Immunoblotting analysis was performed to detect the protein levels of cyclin B1. Representative results are presented in A and quantitative results are shown in B. (C, D) HeLa and MCF‐7 cells stably expressing pCDH and Flag‐MORC2 were synchronized at prometaphase using nocodazole and released into mitosis for the indicated times. Immunoblotting analysis was performed to detect the protein levels of cyclin B1. Representative results are presented in C and the corresponding quantitative results are shown in D. (E, F) HEK293T cells were first transfected with pLVX and HA‐Cdc20 expression vectors and then infected with or without Flag‐MORC2 lentivirus, followed by incubation with or without 100 nM PTX (E) or VCR (F) for 24 h. IP assays using HA‐tagged beads were performed to detect the association between Cdc20 and Bub3/BubR1. (G, H) WT and MORC2‐KO HeLa and MCF‐7 cells were treated with or without 100 nM PTX (G) or VCR (H) for 24 h. IP assays using an anti‐Cdc20 antibody were performed to detect the association between Cdc20 and Bub3/BubR1. (I) Immunoblotting analysis was performed to detect the protein levels of Cdc20 in HeLa and MCF‐7 cells stably expressing pCDH and Flag‐MORC2. (J) Immunoblotting analysis was performed to detect the protein levels of Cdc20 in WT and MORC2‐KO HeLa and MCF‐7 cells. (K, L) WT and MORC2‐KO HeLa and MCF‐7 cells were transfected with siNC or siRNAs targeting Cdc20 and then incubated with or without 100 nM PTX (K) or VCR (L) for 24 h. Immunoblotting analysis was performed to detect the expression levels of H3 pS10.

As previously stated, PTX and VCR inhibit cell replication by disrupting microtubule assembly, thereby promoting mitotic arrest by activating the SAC. Thus, reduced mitotic arrest in response to the drugs while MORC2 exists is likely to result from compromised mitotic checkpoint function. Cyclin B1 protein, a known APC/C downstream substrate, is maintained at a high level due to the ability of activated SAC to inhibit the E3 ubiquitin ligase activity of APC/C. To test whether MORC2 is able to induce SAC inactivation, we investigated the kinetics of cyclin B1 degradation after nocodazole treatment. As expected, cyclin B1 protein was more stable in MORC2‐KO cells (Figure 6A,B), whereas the stability of cyclin B1 protein was decreased after MORC2 overexpression (Figure 6C,D). These results confirmed that MORC2 may compromise SAC activity. It has been shown that some proteins influence SAC function by affecting the stability of the MCC.^14^, ^67^, ^68^ To determine whether MORC2 also compromises SAC activity by affecting the assembly of the MCC, IP analysis was performed to detect the interaction between MCC proteins. Results showed that PTX and VCR treatment significantly increased the interaction between Cdc20 and BubR1/Bub3, two key components of the MCC, suggesting that PTX and VCR activated the SACand promoted the normal assembly of the MCC. However, ectopic expression of MORC2 dramatically reduced the increase in the interaction of exogenous and endogenous Cdc20 with BubR1/Bub3 caused by PTX and VCR (Figure 6E,F and Figure S10A,B). In contrast, MORC2 knockout remarkably enhanced the binding of Cdc20 with BubR1 and Bub3 (Figure 6G,H). These findings indicate that MORC2 impairs the function of the SAC by suppressing the assembly of the MCC.

Interestingly, we also discovered that there seemed to be a negative correlation between the protein levels of MORC2 and Cdc20 according to the results of IP assays (Figure 6G,H). This phenomenon was further validated by examining Cdc20 expression levels upon overexpression or knockout of MORC2. Immunoblotting analysis showed that ectopic expression of MORC2 led to a downregulation (Figure 6I), whereas MORC2 knockout resulted in an upregulation (Figure 6J), of Cdc20 expression levels. Moreover, we observed that the expression levels of MORC2 were decreased after PTX and VCR treatment, while the protein levels of Cdc20 were increased (Figure S10C,D). These findings indicate that MORC2 negatively regulates Cdc20 expression. To address whether MORC2 modulates the proportion of cells arrested in mitosis by inhibiting Cdc20 after treatment with PTX and VCR, we knocked down Cdc20 using siRNAs in MORC2‐KO cells. Results showed that the increase in H3 pS10 expression levels resulting from MORC2 knockout could be reversed by knocking down Cdc20 (Figure 6K,L). Altogether, these results demonstrated that MORC2 is able to compromise the function of the SAC.

### 3.8 MORC2 induces Cdc20 degradation via the ubiquitin‐proteasome pathway

**FIGURE 7:**
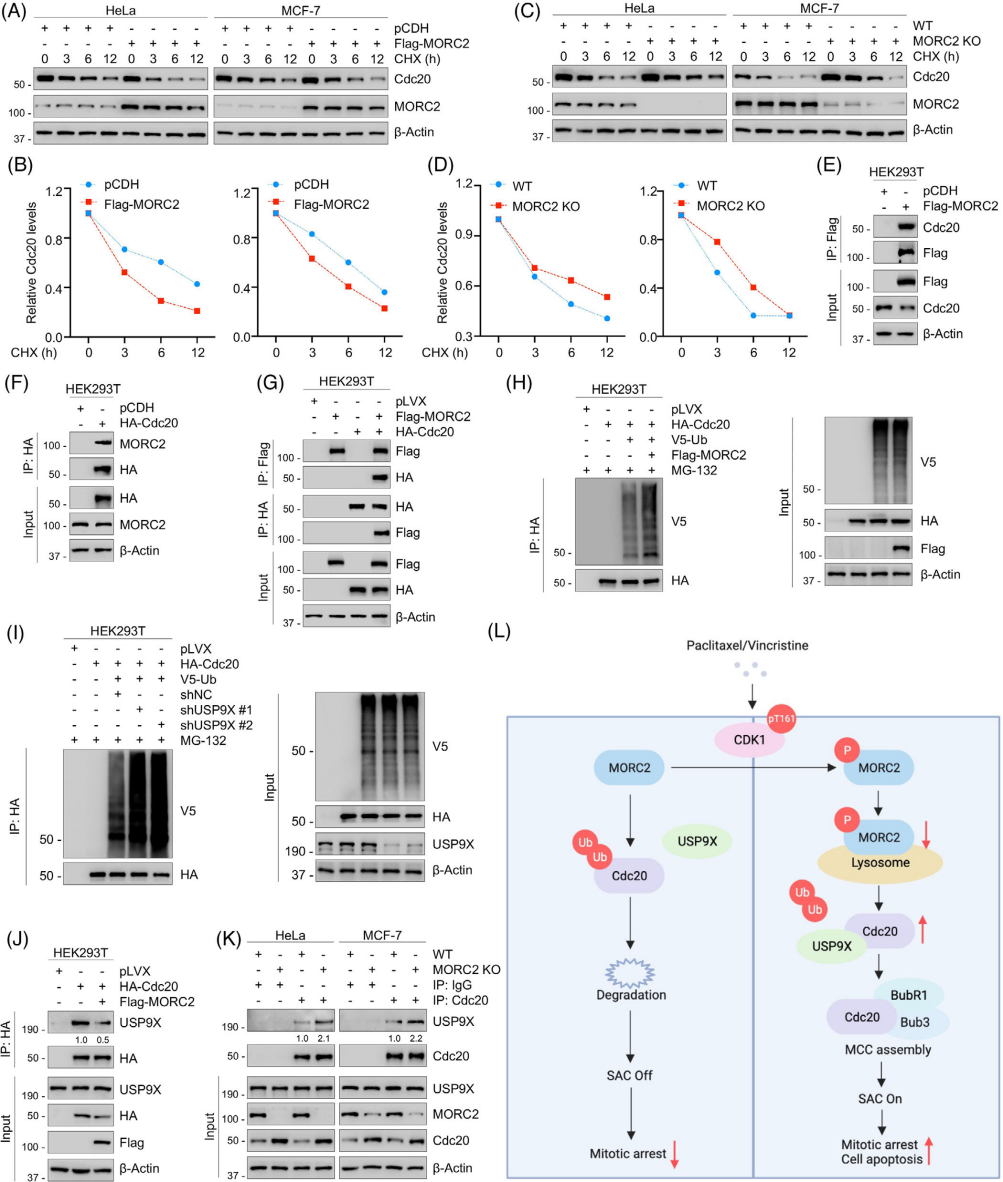
MORC2 induces proteasomal degradation of Cdc20 via blocking the interaction of USP9X with CDC20. (A, B) HeLa and MCF‐7 cells stably expressing pCDH and Flag‐MORC2 were incubated with 100 μg/ml CHX for the indicated times. Immunoblotting analysis was performed to detect the protein levels of Cdc20. Representative results are presented in A and quantitative results are shown in B. (C, D) WT and MORC2‐KO HeLa and MCF‐7 cells were incubated with 100 μg/ml CHX for the indicated times. Immunoblotting analysis was performed to detect the protein levels of Cdc20. Representative results are presented in C and quantitative results are shown in D. (E) HEK293T cells were transfected with pCDH or Flag‐MORC2 expressing vector. IP analysis was performed using Flag‐tagged beads to detect the interaction between MORC2 and Cdc20. (F) HEK293T cells were transfected with pLVX or HA‐Cdc20 expressing vector. IP analysis was performed using HA‐tagged beads to detect the interaction between Cdc20 and MORC2. (G) HEK293T cells were transfected with Flag‐MORC2 and HA‐Cdc20 expressing vectors alone or in combination. IP analysis was performed using Flag‐ or HA‐tagged beads to detect the interaction between MORC2 and Cdc20. (H) HEK293T cells were first co‐transfected with HA‐Cdc20, V5‐ubiquitin (Ub) expression vectors and then infected with or without Flag‐MORC2 lentivirus, followed by incubation with 10 μM MG‐132 for 6 h. IP analysis using HA‐tagged beads was performed to detect the ubiquitination levels of Cdc20. (I) HEK293T cells were first co‐transfected with HA‐Cdc20, V5‐ubiquitin (Ub) expression vectors and then infected with or without shNC and shUSP9X (#1 and #2) lentivirus, followed by incubation with 10 μM MG‐132 for 6 h. IP analysis using HA‐tagged beads was performed to detect the ubiquitination levels of Cdc20. (J) HEK293T cells were first transfected with pLVX and HA‐Cdc20 expression vectors and then infected with or without Flag‐MORC2 lentivirus. IP analysis using HA‐tagged beads was performed to detect the association between Cdc20 and USP9X. (K) IP analysis using control IgG or an anti‐Cdc20 antibody was performed to detect the association between Cdc20 and USP9X in WT and MORC2‐KO HeLa and MCF‐7 cells. (L) The proposed working model.

To investigate whether MORC2 regulates Cdc20 expression at the transcription level, RT‐qPCR assays were carried out. As shown in Figure S11A,B, neither overexpression nor knockdown of MORC2 affected Cdc20 mRNA levels. In contrast, CHX chase assays demonstrated that the half‐life of Cdc20 was shortened in MORC2‐overexpressing cells (Figure 7A,B), whereas the stability of Cdc20 protein was prolonged in MORC2‐KO cells (Figure 7C,D). Based on these findings, we speculated that MORC2 might modulate Cdc20 posttranscriptionally. To validate this notion, the association between MORC2 and Cdc20 was detected by IP assays (Figure 7E–G). We discovered that the proteasome inhibitor MG‐132 led to an accumulation of Cdc20 protein levels, suggesting that Cdc20 is degraded through the ubiquitin‐proteasome pathway (Figure S11C). Furthermore, in vivo ubiquitination analysis showed that MORC2 overexpression dramatically increased Cdc20 ubiquitination levels (Figure 7H). These results collectively demonstrated that MORC2 downregulates Cdc20 through the ubiquitin‐proteasome pathway.

Given that MORC2 is not a putative E3 ubiquitin ligase, it is unlikely that MORC2 directly induces the polyubiquitination of Cdc20. Interestingly, it has been shown that deubiquitinase ubiquitin‐specific peptidase 9 X‐linked (USP9X) could inhibit ubiquitination and degradation of Cdc20, thus reinforcing the SAC.^69^ Thus, we next attempted to verify whether MORC2 regulates the polyubiquitination and stability of Cdc20 through USP9X. IP assays confirmed that endogenous Cdc20 indeed interacted with endogenous USP9X (Figure S11D). Moreover, knockdown of USP9X using shRNAs reduced Cdc20 protein levels (Figure S11E) and simultaneously enhanced Cdc20 ubiquitination levels (Figure 7I). However, we found that neither overexpression nor knockdown of MORC2 affected the protein levels of USP9X (Figure S11F,G). Next, we assessed whether MORC2 modulates the ubiquitination and protein stability of Cdc20 by interfering with the interaction between Cdc20 and USP9X. As expected, IP assays revealed that MORC2 overexpression indeed compromised (Figure 7J), whereas MORC2 knockout enhanced (Figure 7K), the association between Cdc20 and USP9X. Collectively, these results suggest that MORC2 increases the ubiquitination levels of Cdc20 and reduces its protein stability by compromising the interaction between Cdc20 and USP9X. It has been previously reported that ubiquitination and degradation of Cdc20 in mitosis facilitates APC/C‐MCC disassembly, allowing APC/C to form a complex with the newly synthesized Cdc20, leading to a weakened SAC.^67^ Therefore, our results further demonstrated that the presence of MORC2 may compromise the function of the SAC by promoting the ubiquitination and subsequent degradation of Cdc20.

## 4 DISCUSSION

In the present study, several intriguing findings are presented regarding the novel functional and molecular mechanisms of MORC2 downregulation upon PTX and VCR treatment. Briefly, we discovered that PTX and VCR promote the phosphorylation of MORC2 by activating CDK1, resulting in its degradation via the CMA pathway. Downregulation of MORC2 further activates the SAC by stabilizing Cdc20 and promoting the assembly of the MCC, thus contributing to mitotic arrest induced by PTX and VCR (Figure 7L).

First, microtubules are cytoskeletal structures that are critical for maintaining normal cell division. Drugs targeting microtubule proteins and microtubule systems are important components of combination chemotherapy for a large amount of pediatric and adult malignancies. MTAs can be divided into two categories: microtubule‐stabilizing (such as PTX) and microtubule‐destabilizing (such as VCR and vinblastine) agents.^70^, ^71^ However, predicting the responsiveness of patients remains difficult and chemoresistance has become a serious issue in cancer therapy that is linked to poor outcomes, tumour recurrence and metastasis.^72^ To date, the mechanisms of resistance of cancer cells to MTAs are gradually starting to be elucidated. For example, it was reported that SYTL4 contributes to PTX resistance by downregulating microtubule stability.^73^ In addition, aberrant expression of specific microtubule‐associated proteins is strongly relevant to resistance to MTAs.^74^

MORC2, a poorly defined oncoprotein, is markedly upregulated in most tumour tissues and promotes cancer cell growth, metastasis and drug resistance.^32^, ^46^, ^47^, ^56^, ^75^ We report here for the first time that MORC2 knockout contributes to PTX‐ and VCR‐induced mitotic arrest and enhances cancer cellular sensitivity to these MTAs (Figure 5). Thus, MORC2 can be used as a biomarker for PTX and VCR resistance, which has important clinical significance. These results also provide a clue for optimizing the patient selection of these anti‐microtubule therapies and for developing potential combination therapy strategies by targeting MORC2 in combination with PTX or VCR for cancer treatment.

Second, previous studies have revealed that phosphorylation of proteins and protein turnover play an important role in affecting cellular sensitivity to chemotherapeutic drugs. For instance, PTX induces phosphorylation of IRAK1 to activate the IRAK1 pathway, resulting in resistance to PTX.^76^ In our study, we found that MORC2 was phosphorylated by CDK1 in the presence of PTX and VCR, which induced the degradation of MORC2 through the CMA pathway (Figures 2, 3, 4). These results suggest that MORC2 may play a role in influencing cell sensitivity to these two drugs.

In addition, we also demonstrated that phosphorylation and instability of MORC2 induced by PTX and VCR depend on CDK1. It has been shown that CDK1 can be activated by PTX and VCR and regulates cell‐cycle progression by phosphorylating its downstream substrates.^25^ In the presence of PTX and VCR, CDK1 was activated to induce phosphorylation of MORC2 at T717 and T733, thus enhancing the interaction between MORC2 and HSPA8/LAMP2A to reduce MORC2 protein stability (Figures 3 and 4). These results highlight an important role for CDK1 in regulating the biological function of MORC2 through phosphorylation modification. In support of our results, it has been shown that phosphorylation of p300 by CDK1 enhances its degradation and mitotic progression.^77^ However, given the fact that mutation of T717 and T733 residues in MORC2 have not been identified in TCGA and other cancer databases so far, the molecular mechanism of upregulation of MORC2 in cancer, and whether it involves phosphorylation at T717 and T733, remain to be further investigated in the future.

Third, we found that the degradation of MORC2 is essential for SAC activation. MTAs, such as PTX and VCR, have been shown to interfere with the assembly kinetics of microtubules and cause cells to be arrested in mitosis, leading to apoptosis by continuously activating the SAC. The impaired SAC in cancer cells will lead to cells escaping mitotic arrest and avoiding apoptosis, thus resulting in chemo resistance.^78^, ^79^ In addition, downregulation of BRCA1 was found to downregulate BubR1, an essential component of the SAC, thereby leading to PTX resistance in MCF‐7 cells.^80^ Sudo et al.^16^ also confirmed that downregulation of Mad2 and BubR1 abolish SAC function upon PTX treatment, resulting in PTX resistance. Based on these studies, we speculated that MORC2 may affect the sensitivity of cancer cells to PTX and VCR by inhibiting the function of the SAC. Indeed, we demonstrated that MORC2 compromised SAC function by negatively regulating Cdc20 expression levels and inhibiting the formation of the MCC (Figures 6 and 7). Consistently, MORC2 knockout sensitized breast cancer cells to PTX and VCR. In support of our findings, Cdc20 has been found to be stabilized by tumour suppressor DAB2IP, and thus reinforces SAC function.^67^ In addition, SNCG can attenuate the interaction between BubR1 and other MCC proteins, thus destabilizing MCC and compromising SAC function, which ultimately leads to chemoresistance.^14^

When we investigated the mechanism by which MORC2 regulates Cdc20 expression levels, we discovered that MORC2 enhanced Cdc20 ubiquitination by compromising the interaction between Cdc20 and USP9X. Emerging evidence shows USP9X as a key DUB involved in cell‐cycle regulation and chemoresistance. For example, it was reported that USP9X could reinforce the SAC by inhibiting the ubiquitination and degradation of Cdc20.^69^ USP9X also can induce deubiquitylation and stabilization of XIAP, resulting in increased resistance toward MTAs in aggressive B‐cell lymphoma.^81^ These findings further support the functional role of MORC2 in mitotic arrest and SAC function. However, the detailed mechanism by which MORC2 regulates the Cdc20‐USP9X association needs to be elucidated in the future.

In summary, our findings demonstrate that MORC2 has previously unidentified biological functions in regulating mitotic progression and SAC activation in response to MTAs. Therefore, discovering new drugs targeting MORC2 may enhance the effectiveness of MTAssuch as PTX and VCR against human cancer. Our findings also provide novel mechanistic insights into how these MTAs work in cells and may be useful for selecting the appropriate patients to be treated with these chemotherapeutic drugs.

## CONFLICT OF INTEREST STATEMENT

The authors declare no conflict of interest.

## Supporting information

Supporting Information
