# Supplementary material for: Destabilization of microrchidia family CW‐type zinc finger 2 via the cyclin‐dependent kinase 1‐chaperone‐mediated autophagy pathway promotes mitotic arrest and enhances cancer cellular sensitivity to microtubule‐targeting agents
Source: Clin Transl Med. 2023 Mar 26;13(3):e1210. doi: 10.1002/ctm2.1210 (PMC10040724; doi:10.1002/ctm2.1210)
Supplement: Supplementary file 1 — Supporting Information [file CTM2-13-e1210-s001.docx]

**Supplementary information for**

Hu et al. Destabilization of MORC2 via the CDK1-CMA pathway promotes mitotic arrest and enhances cancer cellular sensitivity to microtubule-targeting agents

**The supplementary information includes:**

Supplementary Figures S1-S11

Supplementary Tables S1-S6

**Supplementary figures and figure legends**

**
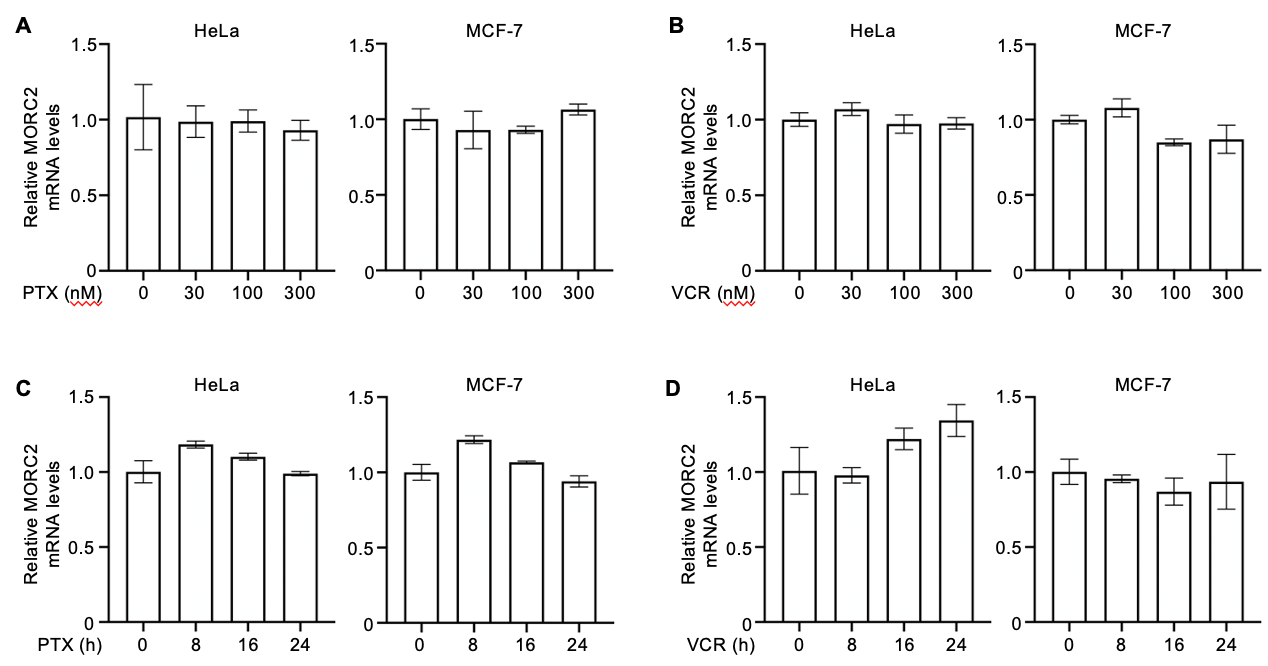
**

**Figure S1. PTX and VCR do not affect the mRNA levels of MORC2**

(A-B) HeLa and MCF-7 cells were treated with indicated doses of PTX (A) or VCR (B) for 24 h. Total RNAs were isolated and subjected to RT-qPCR assays to analyze MORC2 mRNA levels.

(C-D) HeLa and MCF-7 cells were treated with 100 nM PTX (C) or VCR (D) for indicated times. Total RNAs were isolated and subjected to RT-qPCR assays to analyze MORC2 mRNA levels.

**
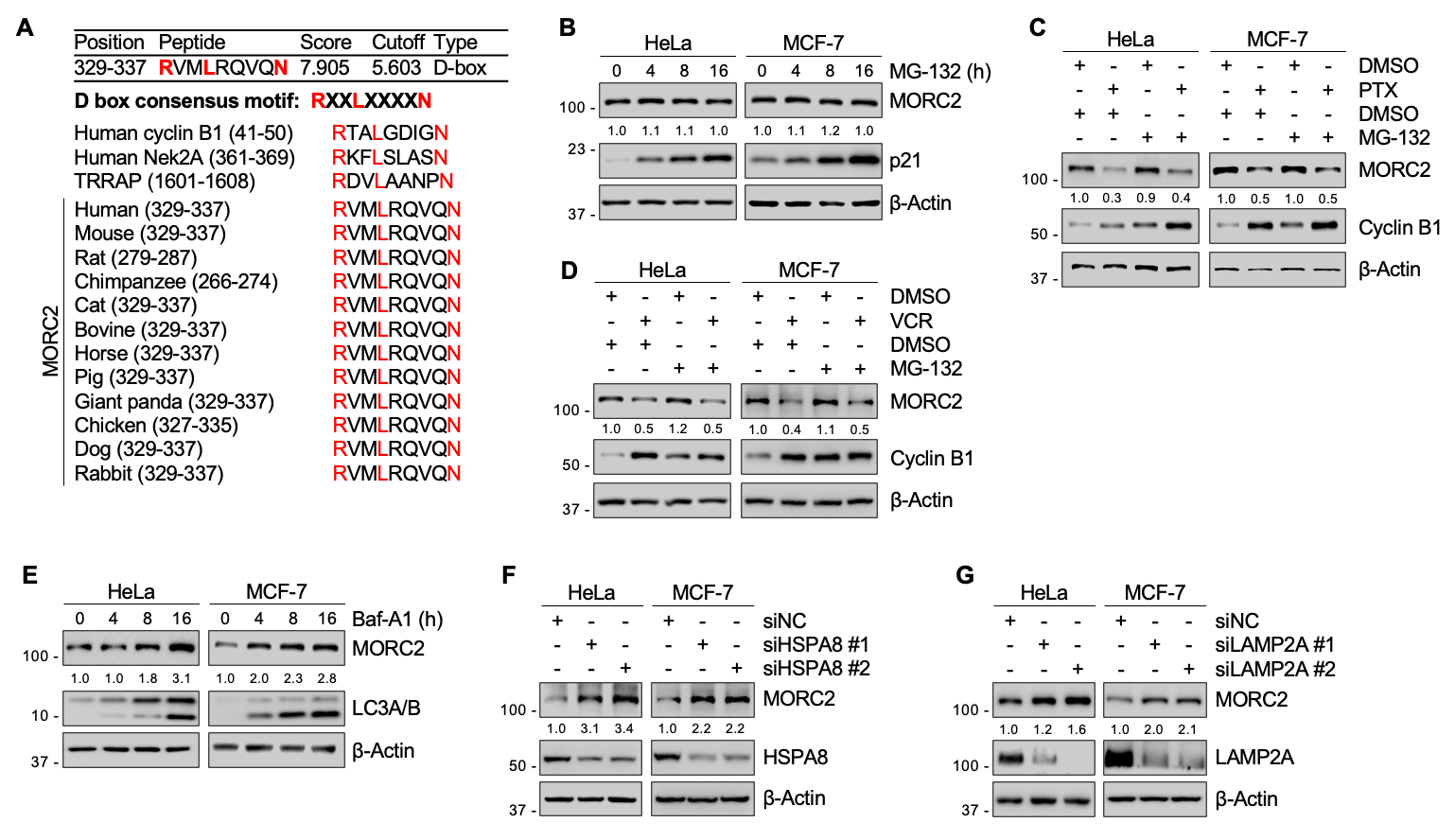
**

**Figure S2. MORC2 is degraded through the CMA pathway**

(A) The presence of a destruction box motif in MORC2.

(B) HeLa and MCF-7 cells were treated with or without 10 μM MG-132 for the indicated times and analyzed by immunoblotting.

(C-D) HeLa and MCF-7 cells were pretreated with or without 10 μM MG-132 for 1 h and then treated with or without 100 nM PTX or VCR for 24 h. Immunoblotting analysis was performed with the indicated antibodies.

(E) HeLa and MCF-7 cells were treated with 50 ng/ml Baf-A1 for indicated times and analyzed by immunoblotting.

(F-G) HeLa and MCF-7 cells were transfected with indicated siRNAs for 48 h and then harvested for immunoblotting with the indicated antibodies.

**
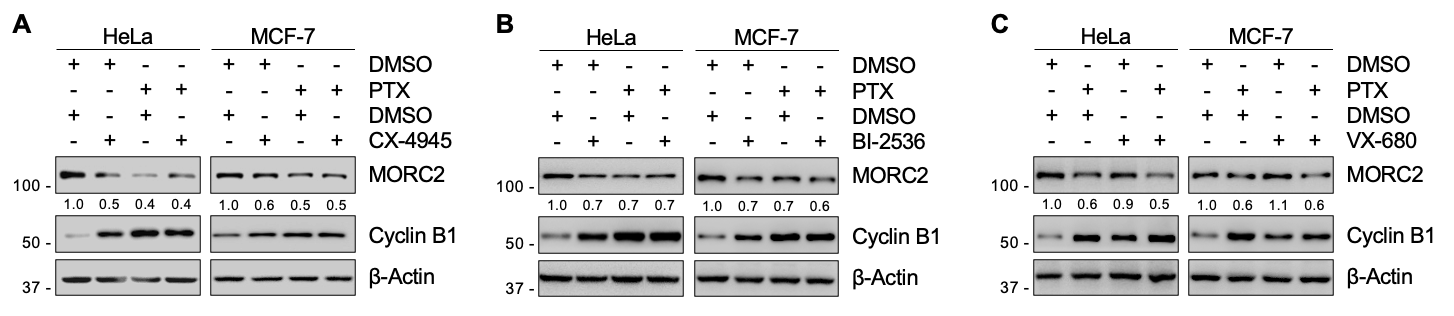
**

**Figure S3. MORC2 degradation induced by PTX is mediated by CDK1**

(A-C) HeLa and MCF-7 cells were pretreated with or without the indicated inhibitors for 1 h and then treated with or without 100 nM PTX for 24 h. Immunoblotting analysis was performed with the indicated antibodies.

**
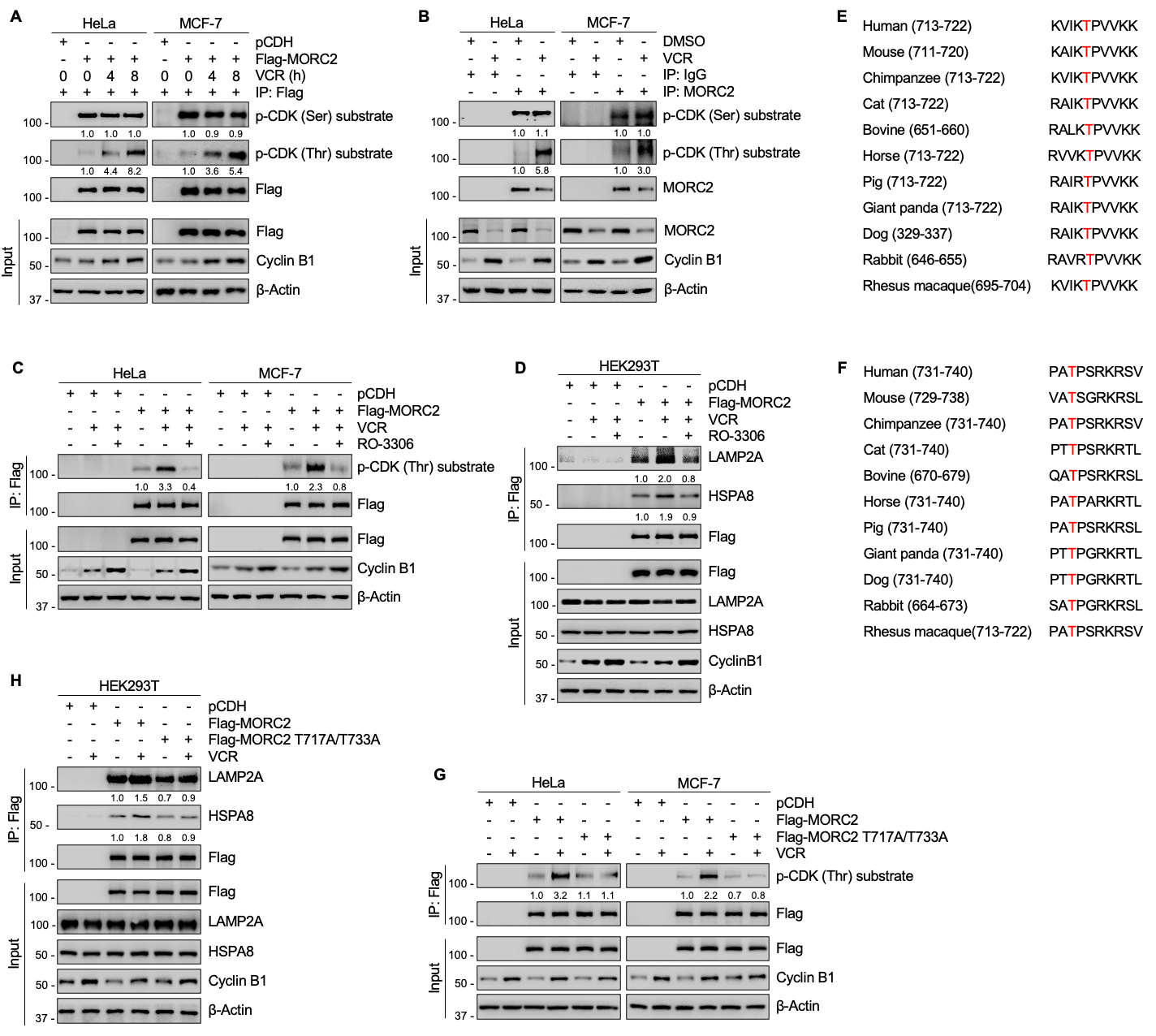
**

**Figure S4. CDK1 phosphorylates MORC2 upon VCR treatment**

(A) HeLa and MCF-7 cells were transfected with pCDH or Flag-MORC2 expression vector. After 48 h of transfection, cells were treated with 100 nM VCR for the indicated times and then subjected to IP and immunoblotting analyses.

(B) HeLa and MCF-7 cells were treated with or without 100 nM VCR for 24 h. Cellular lysates were then subjected to IP analysis using an anti-MORC2 antibody and then analyzed by immunoblotting.

(C) HeLa and MCF-7 cells were transfected with pCDH or Flag-MORC2 expression vector. After 48 h of transfection, cells were pretreated with 5 µM RO-3306 for 1 h and incubated with or without 100 nM VCR for 24 h. Cellular lysates were harvested and subjected to IP and immunoblotting analyses.

(D) HEK293T cells were transfected with pCDH or Flag-MORC2 expression vector. After 48 h of transfection, cells were pretreated with 5 µM RO-3306 for 1 h and incubated with or without 100 nM VCR for 24 h. IP and immunoblotting analyses were performed with the indicated antibodies.

(E) Alignment of MORC2 T717 phosphorylation site across different species.

(F) Alignment of MORC2 T733 phosphorylation site across different species.

(G) HeLa and MCF-7 cells were transfected with indicated expression vectors. After 48 h of transfection, cells were treated with or without 100 nM VCR for 24 h and subjected to IP assays with anti-Flag beads, followed by immunoblotting analysis.

(H) HEK293T cells were transfected with indicated expression vectors. After 48 h of transfection, cells were treated with or without 100 nM VCR for 24 h and subjected to IP assays followed by immunoblotting analysis.

**
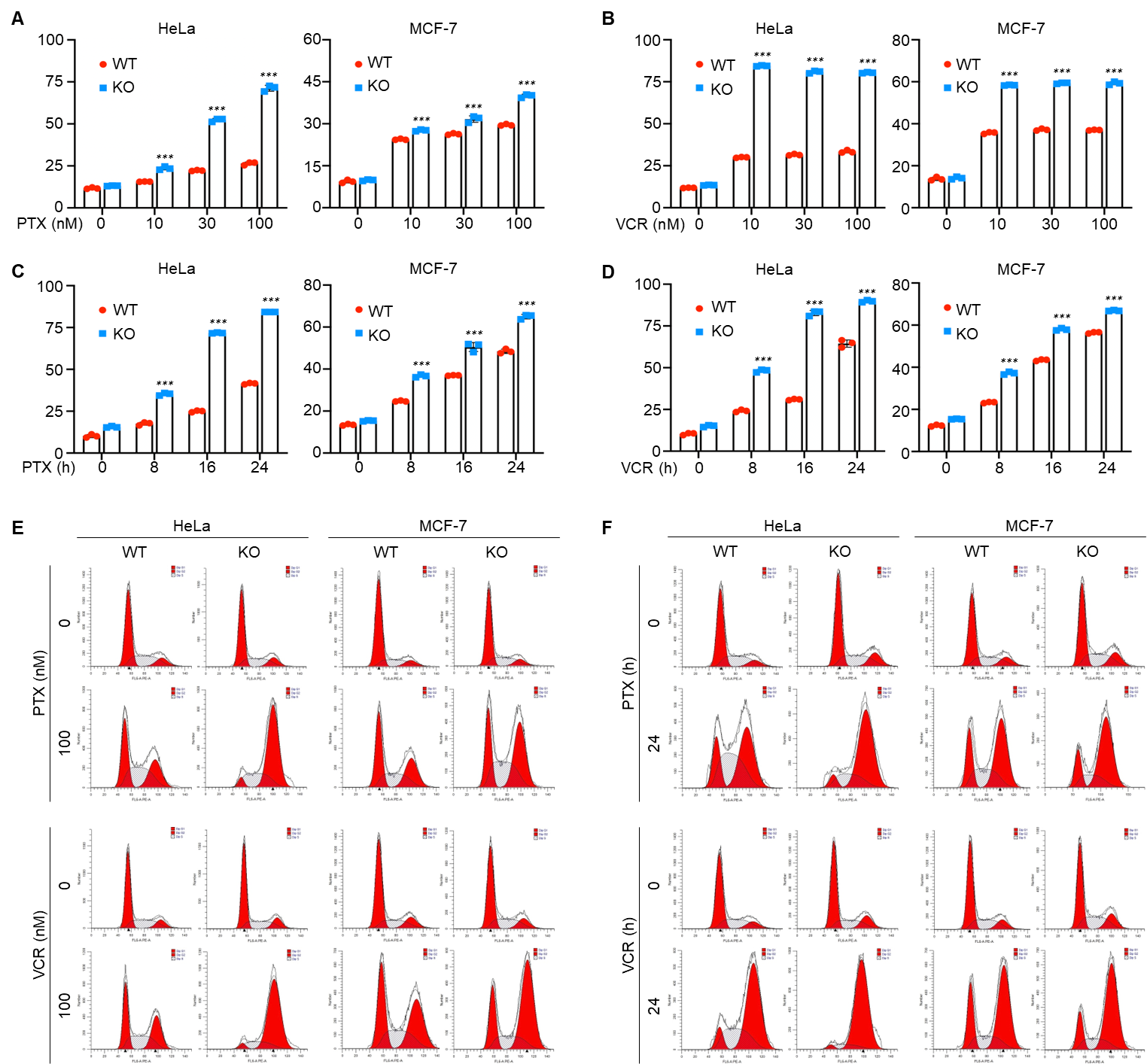
**

**Figure S5. Depletion of MORC2 enhances the effects of PTX and VCR on mitosis arrest**

(A-B) HeLa and MCF-7 cells were treated with the indicated doses of PTX (A) or VCR (B) for 14 h. Cell-cycle distribution was determined by FACS analysis.

(C-D) HeLa and MCF-7 cells were treated with 30 nM PTX (C) or VCR (D) for the indicated times. Cell-cycle distribution was determined by FACS analysis.

(E-F) Representative images of FACS analysis are shown.

**
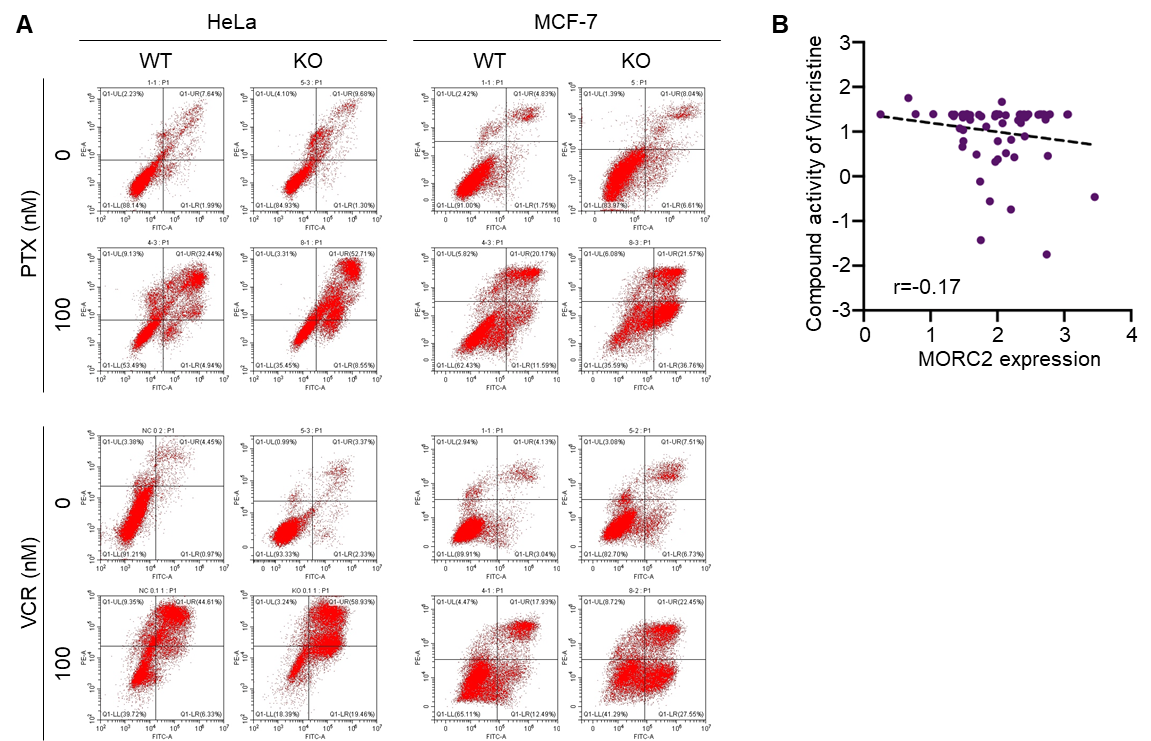
**

**Figure S6. Depletion of MORC2 sensitizes cells to PTX and VCR**

(A) HeLa and MCF-7 cells were treated with the indicated doses of PTX and VCR for 48 h. Both adherent and floating cells were harvested and subjected to FACS analysis. Representative images of FACS analysis are shown in A.

(B) The correlation between MORC2 expression levels and drug activity of VCR from the CellMiner database.

**
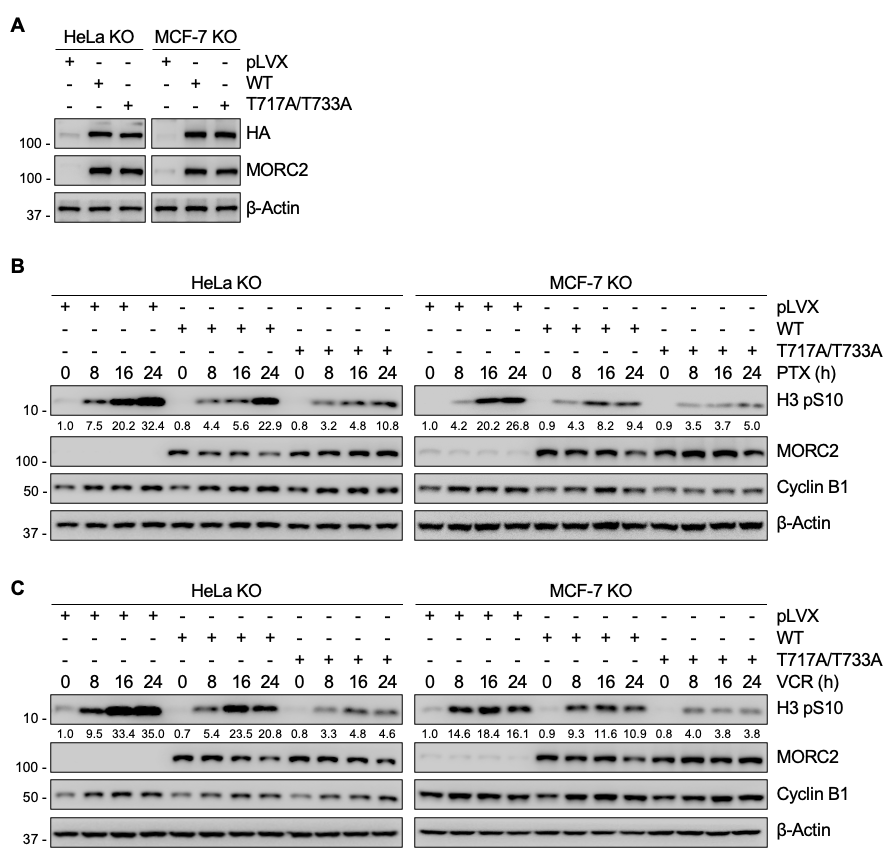
**

**Figure S7. T717A/T733A mutant MORC2 suppresses PTX- and VCR-induced mitotic arrest**

(A) WT and T717A/T733A mutant HA-MORC2 were reinduced into MORC2-KO HeLa and MCF-7 cells through lentiviral infection. Expression levels of reconstituted MORC2 were validated by immunoblotting.

(B-C) MORC2-KO HeLa and MCF-7 cells stably expressing pLVX, WT, and T717A/T733A mutant MORC2 were treated with 100 nM PTX (B) or VCR (C) for the indicated times. Cellular lysates were subjected to immunoblotting assays with the indicated antibodies.

**
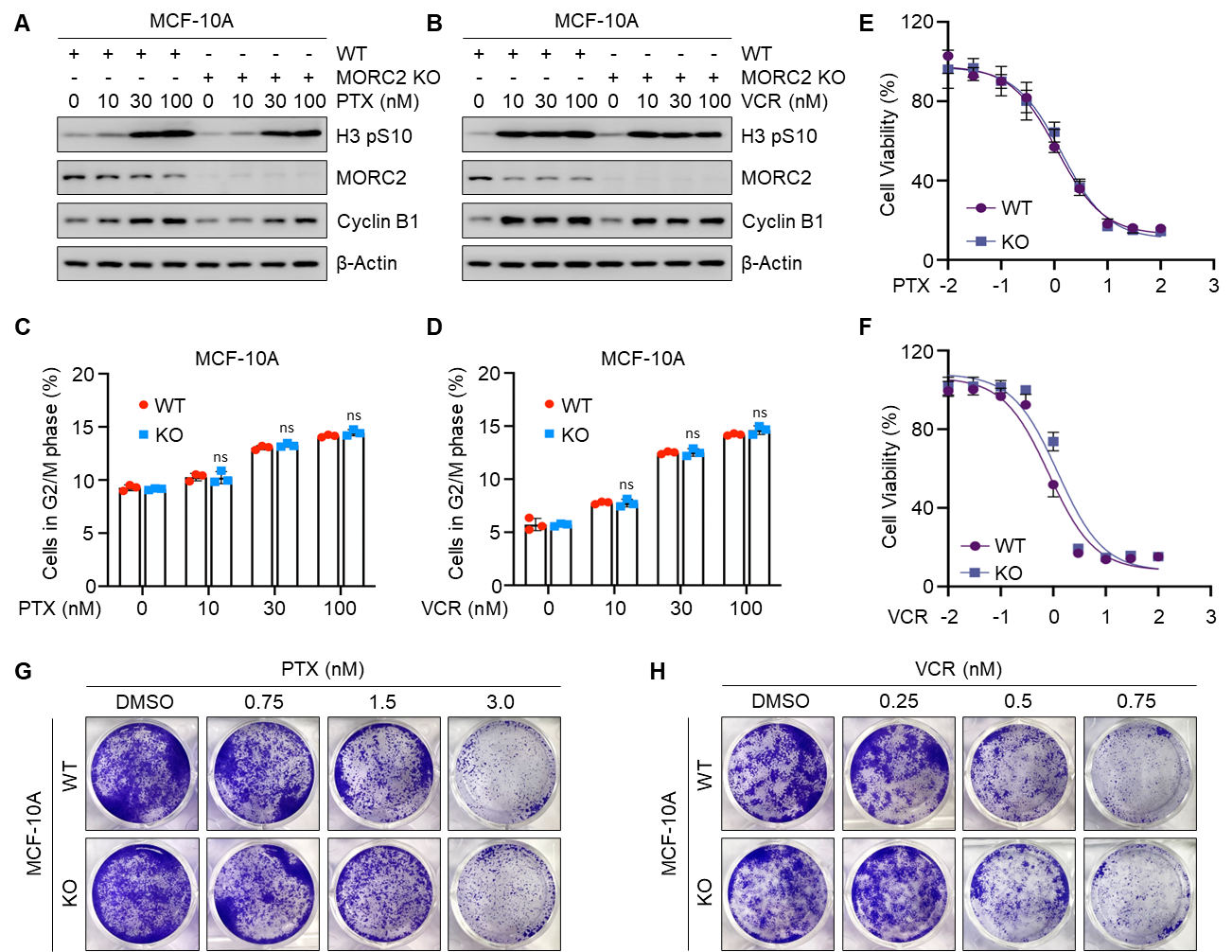
**

**Figure S8. Depletion of MORC2 does not enhance the effects of PTX and VCR in MCF-10A cells**

(A-B) WT and MORC2-KO MCF-10A cells were treated with indicated doses of PTX (A) or VCR (B) for 24 h. Immunoblotting analysis was performed to detect the protein levels of H3 pS10.

(C-D) WT and MORC2-KO MCF-10A cells were treated with indicated doses of PTX (C) or VCR (D) for 24 h. Cell-cycle distribution was determined by FACS analysis.

(E-F) WT and MORC2-KO MCF-10A cells were treated with increasing doses of PTX (E) or VCR (F) for 72 h, and then subjected to CCK-8 assays. Quantitative results are represented as mean ± S.D. as indicated (n=4).

(G-H) WT and MORC2-KO MCF-10A cells were treated with PTX or VCR at the indicated doses and sujected to colony formation assay. After 10 days of treatment, survival colonies were stained with 1% crystal violet. Representative images of survival colonies are shown. **
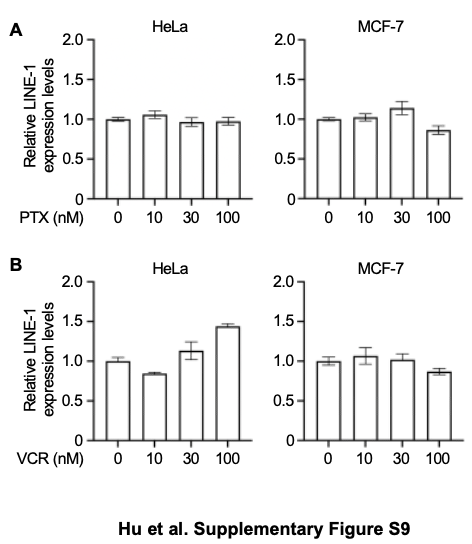
**

**Figure S9. PTX and VCR do not affect LINE-1 transcription**

(A-B) HeLa and MCF-7 cells were treated with the indicated doses of PTX (A) or VCR (B) for 24 h. Total RNAs were isolated and subjected to RT-qPCR assays to analyze LINE-1 expression levels.

**
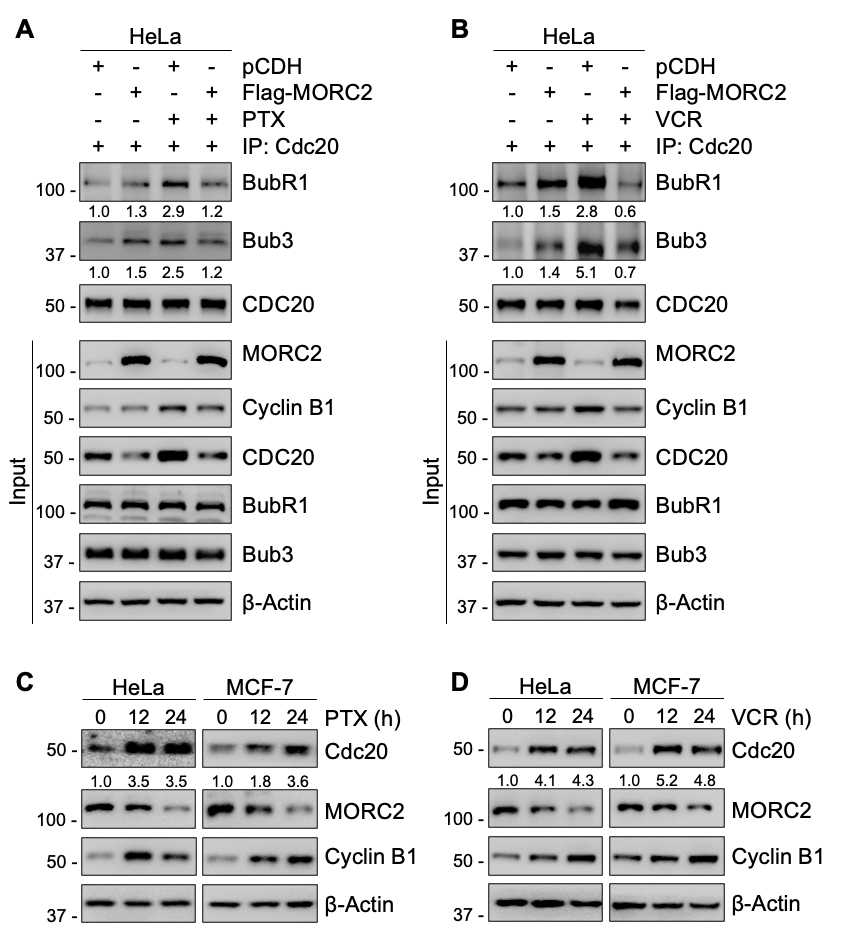
**

**Figure S10. MORC2 compromises the SAC function**

(A-B) HeLa cells were treated with or without 100 nM PTX (A) or VCR (B) for 24 h and then subjected to IP assays with an anti-Cdc20 antibody, followed by immunoblotting analysis.

(C-D) HeLa and MCF-7 cells were treated with 100 nM PTX (C) or VCR (D) for the indicated times. Cellular lysates were subjected to immunoblotting assays with the indicated antibodies.

**
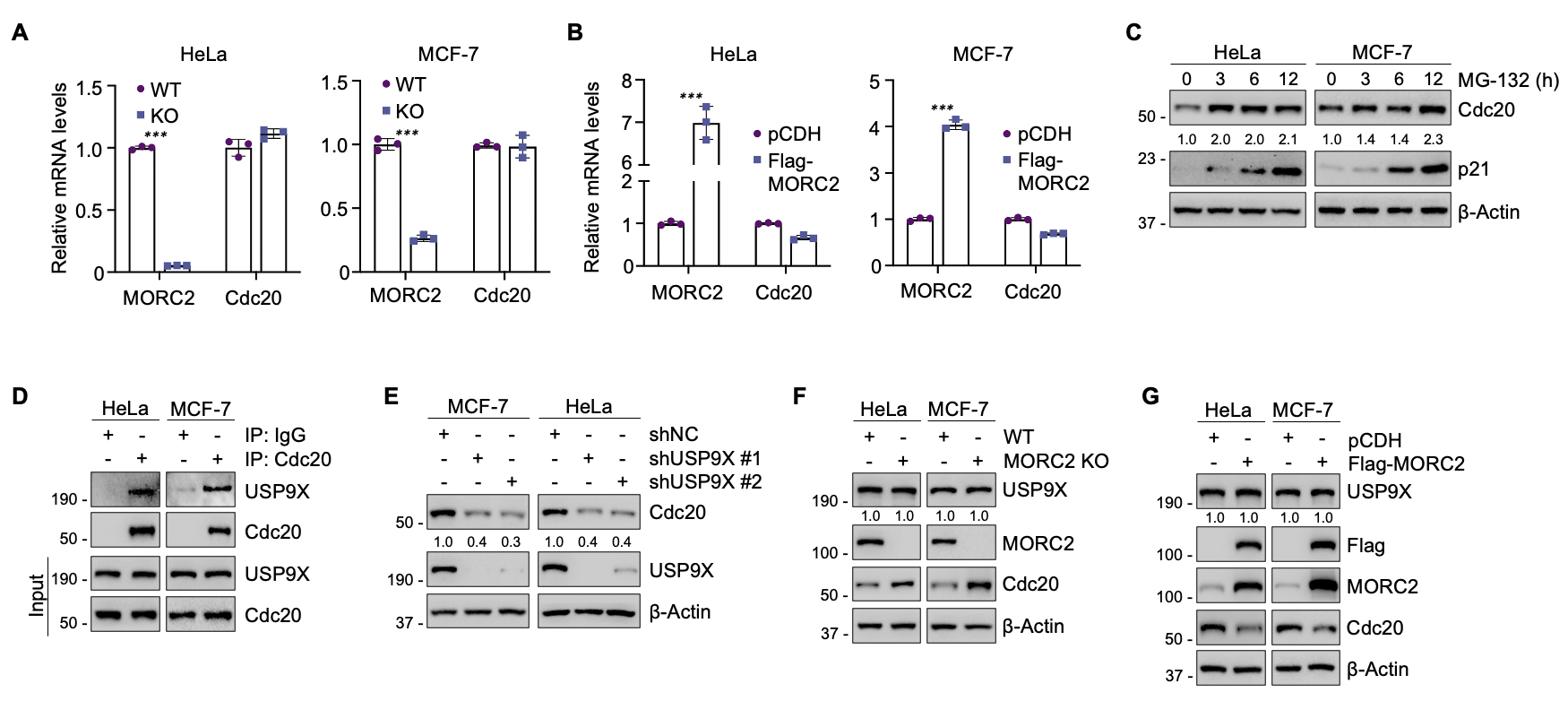
**

**Figure S11. MORC2 induces Cdc20 degradation via the ubiquitin-proteasome pathway**

(A) WT and MORC2-KO HeLa and MCF-7 cells were subjected to RT-qPCR analysis of the mRNA levels of MORC2 and Cdc20.

(B) HeLa and MCF-7 cells stably expressing pCDH and Flag-MORC2 were subjected to RT-qPCR analysis of the mRNA levels of MORC2 and Cdc20.

(C) HeLa and MCF-7 cells were incubated with 10 μM MG-132 for the indicated times. Cellular lysates were collected and analyzed by immunoblotting.

(D) Cellular lysates of HeLa and MCF-7 cells were collected and subjected to IP assays with an anti-Cdc20 antibody or control IgG, followed by immunoblotting analysis.

(E) HeLa and MCF‐7 cells stably expressing shNC and shUSP9X (#1 and #2) were subjected to immunoblotting analysis with the indicated antibodies.

(F) HeLa and MCF-7 cells stably expressing pCDH and Flag-MORC2 were subjected to immunoblotting analysis with the indicated antibodies.

(G) WT and MORC2-KO HeLa and MCF-7 cells were subjected to immunoblotting analysis with the indicated antibodies.

**Supplementary tables**

**Table S1. Chemical inhibitors used in this study**

| **Inhibitors** | **Vendors** | **Cat#** |
| --- | --- | --- |
| Paclitaxel | Selleck | S1150 |
| Vincristine | Selleck | S1241 |
| MG-132 | Selleck | S2691 |
| Bafilomycin A1 | Selleck | S1413 |
| Thymidine | Selleck | S4803 |
| Nocodazole | Selleck | S2775 |
| Cycloheximide | CST | 2112S |
| RO-3306 | Selleck | S7747 |
| VX-680 | Selleck | S1048 |
| BI-2536 | Selleck | S1109 |
| Protease inhibitor cocktail | Bimake | B14002 |
| Phosphatase inhibitor cocktail | Bimake | B15001 |

**Table S2. Information for the expression vectors used in this study**

| **Plasmids** | **Sources** | **Vectors** |
| --- | --- | --- |
| Myc-DDK-MORC2 | Origene (RC200518) | pCMV6-Entry |
| Flag-MORC2 | Subcloned | pCDH-CMV-MCS-EF1-Puro |
| Flag-MORC2-T582A | Subcloned | pCDH-CMV-MCS-EF1-Puro |
| Flag-MORC2-T717A | Subcloned | pCDH-CMV-MCS-EF1-Puro |
| Flag-MORC2-T733A | Subcloned | pCDH-CMV-MCS-EF1-Puro |
| Flag-MORC2-T717A/T733A | Subcloned | pCDH-CMV-MCS-EF1-Puro |
| HA-Cdc20 | Subcloned | pLVX-IRES-neo |
| Lenti-CAS9 | Addgene | LentiCas9-Blast |
| Lenti-guide | Addgene | LentiGuide-Puro |

**Table S3. Primers used for molecular cloning of expression vectors**

| **Plasmids** | **Primers** | **Sequences** |
| --- | --- | --- |
| pCDH-Flag-MORC2 | Forward | ACCTCCATAGAAGATTCTAGAGCCACCATGCTTTGCTTTTTGGATGATGGAG |
|  | Reverse | GATCCATTTAAATTCGAATTCTTACTTATCGTCGTCATCCTTGTAATC |
| PCDH-Flag-MORC2-T582A | Forward | TTCAGAAAACCGCCCCCATCCGCTC |
|  | Reverse | GAGCGGATGGGGGCGGTTTTCTGAA |
| PCDH-Flag-MORC2-T717A | Forward | AAGTCATCAAGGCCCCAGTGGTGAA |
|  | Reverse | TTCACCACTGGGGCCTTGATGACTT |
| PCDH-Flag-MORC2-T733A | Forward | ATCAAACTCTCCCCGGCTGCCCCTAGTCGGAA |
|  | Reverse | TTCCGACTAGGGGCAGCCGGGGAGAGTTTGAT |
| HA-Cdc20 | Forward | GGATCTATTTCCGGTGAATTCGCCACCATGGCACAGTTCGCGTTCGAG |
|  | Reverse | GGGATCCGCGGCCGCTCTAGATTAAGCGTAGTCTGGGACGTCGTATGGGTAGCGGATGCCTTGGTGGATGAG |

**Table S4. The sequences of siRNAs used in this study**

| **siRNAs** | **Orientations** | **Sequences (5’-3’)** |
| --- | --- | --- |
| siNC | Sense | TATGCCGCTGTGCTCTAT |
|  | Antisense | TTCTTCACCTCCTGCTCC |
| siLAMP2A #1 | Sense | CGCUAUGAAACUACAAAUATT |
|  | Antisense | UAUUUGUAGUUUCAUAGCGTT |
| siLAMP2A #2 | Sense | GCUCUACUUAGACUCAAUATT |
|  | Antisense | UAUUGAGUCUAAGUAGAGCTT |
| siHSPA8 #1 | Sense | GCAAAGAAUCAAGUUGCAATT |
|  | Antisense | UUGCAACUUGAUUCUUUGCTT |
| siHSPA8 #2 | Sense | GCUGUUGUCCAGUCUGAUATT |
|  | Antisense | UAUCAGACUGGACAACAGCTT |
| siCDK1#1 | Sense | GAUCAACUCUUCAGGAUUUTT |
|  | Antisense | AAAUCCUGAAGAGUUGAUCTT |
| siCDK1#2 | Sense | GAUGUAGCUUUCUGACAAAAA |
|  | Antisense | UUUUUGUCAGAAAGCUACAUC |
| siCdc20#1 | Sense | CGGAAGACCUGCCGUUACATT |
|  | Antisense | UGUAACGGCAGGUCUUCCGTT |
| siCdc20#2 | Sense | ACGACAUUUGGCCAGUGGUGGUAAU |
|  | Antisense | AUUACCACCACUGGCCAAAUGUCGU |

**Table S5. Primers for qPCR analysis**

| **Genes** | **Primers** | **Sequences** |
| --- | --- | --- |
| GAPDH | Forward | GGAGCGAGATCCCTCCAAAAT |
|  | Reverse | GGCTGTTGTCATACTTCTCATGG |
| MORC2 | Forward | AGTACGGGAATGGGTTAAAATCG |
|  | Reverse | GAGGCACGTCATGGTGTCT |
| Cdc20 | Forward | GCACAGTTCGCGTTCGAGA |
|  | Reverse | CTGGATTTGCCAGGAGTTCGG |

**Table S6. Information for primary antibodies used in this study**

| **Antibodies** | **Vendors** | **Cat#** | **Species** |
| --- | --- | --- | --- |
| MORC2 | Bethyl | A300-149 | Rabbit monoclonal |
| MORC2 (for IF) | Novus | NBP1-89295 | Rabbit polyclonal |
| Cyclin A2 | CST | 4656P | Mouse monoclonal |
| Cyclin E2 | CST | 4132P | Rabbit monoclonal |
| Cyclin B1 | Abcam | ab32053 | Rabbit monoclonal |
| β-Actin | CST | 3700S | Mouse monoclonal |
| α-Tubulin (for IF) | CST | 3873S | Mouse monoclonal |
| LC3A/B | CST | 12741 | Rabbit monoclonal |
| HSPA8 | Abcam | ab51052 | Rabbit monoclonal |
| LAMP2A | Abcam | ab125068 | Rabbit monoclonal |
| LAMP2A (for IF) | Abcam | ab18528 | Rabbit polyclonal |
| Flag | Sigma | F3165 | Mouse monoclonal |
| 21 Waf1/Cip1 | CST | 2947S | Rabbit monoclonal |
| CDK1 | CST | 9116S | Mouse monoclonal |
| Phospho-CDK1 T161 | Abcam | ab201008 | Rabbit monoclonal |
| Phospho-CDK1 Y15 | CST | 9111P | Rabbit polyclonal |
| Phospho-CDK Substrate Motif [(K/H)pSP] | CST | 9477 | Rabbit monoclonal |
| Phospho-CDK Substrate [pTPXK] (D9V5N) | CST | 14371 | Rabbit monoclonal |
| Phospho-Histone H3 (Ser10) | CST | 3377S | Rabbit monoclonal |
| Cdc20 | CST | 14866 | Rabbit monoclonal |
| Bub3 | Abcam | ab133699 | Rabbit monoclonal |
| BubR1 | Abcam | ab54894 | Mouse monoclonal |
| HA | CST | 3724S | Rabbit monoclonal |
| V5 | CST | 13202S | Rabbit monoclonal |
